# Mechanical constraints to cell-cycle progression in a pseudostratified epithelium

**DOI:** 10.1016/j.cub.2022.03.004

**Published:** 2022-03-25

**Authors:** Sophie Hecht, Gantas Perez-Mockus, Dominik Schienstock, Carles Recasens-Alvarez, Sara Merino-Aceituno, Matthew B. Smith, Guillaume Salbreux, Pierre Degond, Jean-Paul Vincent

**Affiliations:** 1 The Francis Crick Institute, London NW1 1AT, UK; 2 Imperial College London, Department of Mathematics, London SW7 2AZ, UK; 3 University of Vienna, Faculty of Mathematics, Oskar-Morgenstern-Platz 1, Wien 1090, Austria; 4 University of Sussex, Department of Mathematics, Falmer BN1 9RH, UK

## Abstract

As organs and tissues approach their normal size during development or regeneration, growth slows down, and cell proliferation progressively comes to a halt. Among the various processes suggested to contribute to growth termination,^1–10^ mechanical feedback, perhaps via adherens junctions, has been suggested to play a role.^11–14^ However, since adherens junctions are only present in a narrow plane of the subapical region, other structures are likely needed to sense mechanical stresses along the apical-basal (A-B) axis, especially in a thick pseudostratified epithelium. This could be achieved by nuclei, which have been implicated in mechanotransduction in tissue culture.^15^ In addition, mechanical constraints imposed by nuclear crowding and spatial confinement could affect interkinetic nuclear migration (IKNM),^16^ which allows G2 nuclei to reach the apical surface, where they normally undergo mitosis.^17–25^ To explore how mechanical constraints affect IKNM, we devised an individual-based model that treats nuclei as deformable objects constrained by the cell cortex and the presence of other nuclei. The model predicts changes in the proportion of cell-cycle phases during growth, which we validate with the cell-cycle phase reporter FUCCI (Fluorescent Ubiquitination-based Cell Cycle Indicator).^26^ However, this model does not preclude indefinite growth, leading us to postulate that nuclei must migrate basally to access a putative basal signal required for S phase entry. With this refinement, our updated model accounts for the observed progressive slowing down of growth and explains how pseudostratified epithelia reach a stereotypical thickness upon completion of growth.

## Results and Discussion

### Nuclear arrangement in *Drosophila* wing imaginal discs

**Figure 1:**
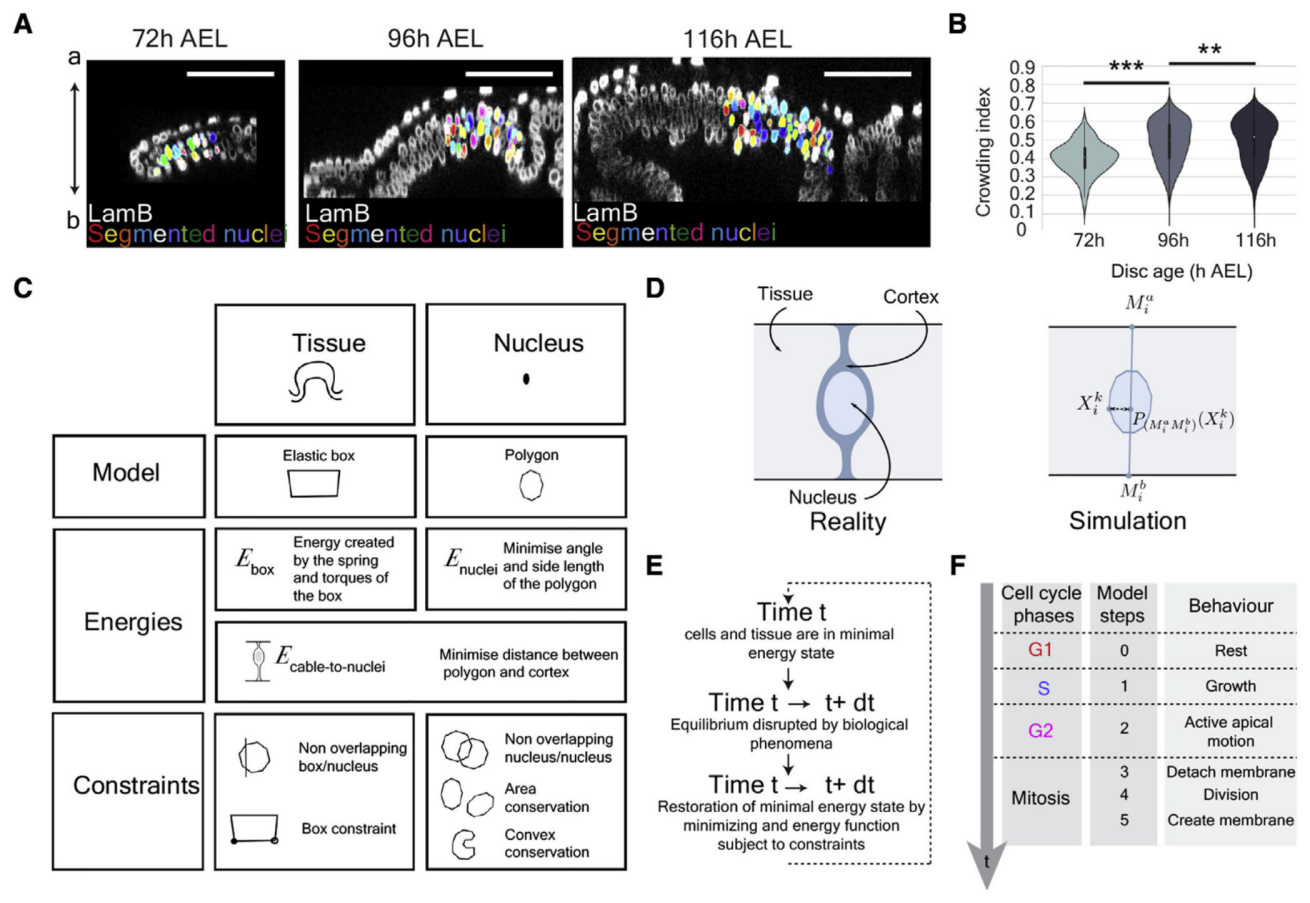
Simulating interkinetic nuclear migration (IKNM) in a confined space (A) Optical cross section of the wing discs shown in Figure S1A. Individual segmented nuclei have been colored randomly. Scale bars represent 50 μm. (B) Distribution of crowding indices in 72, 96, and 116 h AEL wing discs (nuclei located at the border of the segmented area were excluded; see STAR Methods for more details. 72 h AEL: 4 discs, 490 nuclei. 96 h AEL: 4 discs, 1,968 nuclei. 116 h AEL: 3 discs, 5,221 nuclei). Wilcoxon rank-sum statistic test for two samples was performed in (D and H). ** p < 0.01, *** p < 0.001. (C) Overview of the model’s main elements. The edges of the disc (including the apical and basal surfaces) are represented by an elastic box and the nuclei by polygons. The natural curvature of imaginal discs, as seen in Figure 1B, was ignored for simplicity. The energies and constraints of the model are listed. (D) Nuclei were represented as 20-sided deformable polygons, allowing a realistic representation, while limiting computational costs. The constraining effect of the cell cortex was represented by a cable tethered to the apical and basal sides. (E) Iterative progression from one minimal energy state (at time t) to the next (at time t + dt). (F) Behavior of nuclei during the different phases of the cell cycle. Unless stated differently, the duration of the S and G1 phases was defined a priori, whereas the duration of G2 was an output of the model. See also Figure S1.

To evaluate the constraints that nuclei experience during the growth of a pseudostratified epithelium, we first performed detailed morphometric analysis of wing imaginal discs of *Drosophila*, which are epithelial structures that are set aside in the embryo^27^ before undergoing massive growth during larval stages.^28^ We quantified the positions and morphological features of several thousand nuclei, using anti-laminB as a marker, in cleared wing imaginal discs at 72, 96, and 120 h after egg laying (AEL) (Figures 1A, S1A, and S1B; STAR Methods). This showed that, with age, nuclei occupy an increasingly thicker span of the apical-basal (A-B) axis, with 75% of nuclei spread over 10 μm at 72 h AEL, 15 μm at 96 h AEL, and 20 μm at 120 h AEL (Figures S1C and S1D). Therefore, the epithelium grows in thickness as well as in surface area, as shown also by Mao and colleagues.^16^ This is accompanied by increased nuclear crowding, as quantified by the proportion of space surrounding individual nuclei that is occupied by other nuclei (Figures 1B and S1E). We also observed that nuclei became more rounded (quantified by V/lmax, the ratio between volume and largest dimension) between 96 and 116 h, although not during the earlier 72–96 h period (Figure S1F). Therefore, our morphometric analysis and the work of Kirkland et al.^16^ suggest that nuclei find themselves in an evolving mechanical environment during disc growth. We next investigated *in silico* on how this could impact IKNM and hence cell-cycle progression.

### Modeling interkinetic nuclear migration

We opted for an individual-based model to describe dividing nuclei in a confined space because it allowed us to readily incorporate established features of cell-cycle progression in a pseudostratified epithelium. Since it is challenging to model 3D deformable objects, we decided to represent nuclei as 2D objects evolving within a 2D elastic box (Figure 1C; Methods S1). To account for deformability, nuclei were modeled as 20-sided polygons with variable angles and side lengths. In real life, nuclei are confined within the cell membrane, which, in pseudostratified epithelia, maintains a connection to both the apical and basal surfaces, thus preventing nuclei from straying too far laterally. The cell membrane and associated cortex are also expected to exert a squeezing force orthogonal to the A-B axis. These effects were modeled with an energy that minimizes the distance between a virtual apical-to-basal cable and all the polygon’s vertices (Figure 1D). This will be referred to as the cable-to-nuclei energy. The basal anchor of each cable was allowed to move along the basal surface to allow nuclei to move past each other. To calculate the total energy of the system, we considered three features: the elasticity of the box, the deformation of all the nuclei, and the cable-to-nuclei energy. Additional constraints were introduced (1) to prevent nuclei from overlapping with each other or with the box, (2) to ensure surface area conservation and nuclear convexity, and (3) to limit excessive deformation and movement of the box. These energies and constraints allowed us to formally define a minimization problem (see Methods S1). Thus, at any time t, the shape of the box and the location and shape of the nuclei are a solution of this minimization problem; the system is at a minimal energy state and fulfills all constraints. This state was then disrupted by the growth and movement of individual nuclei, and a new minimization cycle was used to compute the next equilibrium at time t + dt (Figure 1E).

We next incorporated specific assumptions to account for the activities known to be associated with various phases of the cell cycle (Figure 1F). In pseudostratified epithelia, nuclei must migrate to the apical surface to undergo mitosis. The mechanistic basis of this requirement is unclear,^17,20,29^ but it is considered to be an essential feature of cell-cycle progression in pseudostratified epithelia (assumption A1). In accordance with previous findings,^21,22,25,30–32^ we assume that the apical-ward movement of G2 nuclei is an active process, probably driven by actomyosin.^21,22^ This was implemented by two forces. First, we introduced a spring connecting the center of mass of the nucleus to the apical anchor point of the apical-to-basal cable. The rest length of this spring was set to zero but with the pulling force inactivated as soon as the edge of the nucleus reaches the apical surface. The second force is governed by a gradient flow energy (see Methods S1) that prevents large movements of nuclei in a single iteration. Since live imaging suggests the existence of a narrow apical region where only mitotic nuclei can enter,^20,21^ we incorporated in the model an apical zone that repels non-mitotic nuclei (see Methods S1). *In vivo*, as nuclei enter this zone, they round up,^33–35^ a process that we implemented by inactivating the cable-to-nuclei energy. Upon completion of nuclear division, a new cell membrane must be generated. In some cases, this is achieved by equal division of the mother cell membrane.^36^ However, it is also observed that one daughter cell maintains the apical and basal connections of the mother while the other daughter grows new extensions that reach the apical and basal surface of the epithelium.^23,37^ We have implemented a similar activity in our simulation by allowing one of the daughters (chosen randomly) to re-establish contacts within 6 or 12 min after mitosis (see Methods S1). As soon as anchor points are re-established, nuclei are allowed to commence their basal-ward descent, which we considered to be passive, under the influence of other nuclei^25,30,38^ (assumption A2). Following mitosis, nuclear volume must obviously grow before another mitosis takes place. Work with cultured cells has suggested that nuclear regrowth can occur during G1 and S.^39–41^ Here, for simplicity, we specified that nuclei double in volume during the S phase only (assumption A3). We now consider the duration of cell-cycle phases. In our initial set of simulations, the duration of S and G1 was specified a priori, with that of G2 being an output of the model. Based on previous estimates^42^ (see Methods S1), we set S phase to last 8 ± 2 h, while G1 was set to last from 2 h at the onset of the simulation (to mimic the situation in young discs) to 10 h at the end (as observed in old discs)^42^ (assumption A4). In subsequent simulations (described in the section entitled “a basal signal could impose a second gate to cell-cycle progression”), neither G2 nor G1 was preassigned.

### The model predicts that crowding affects IKNM and cell-cycle progression

**Figure 2:**
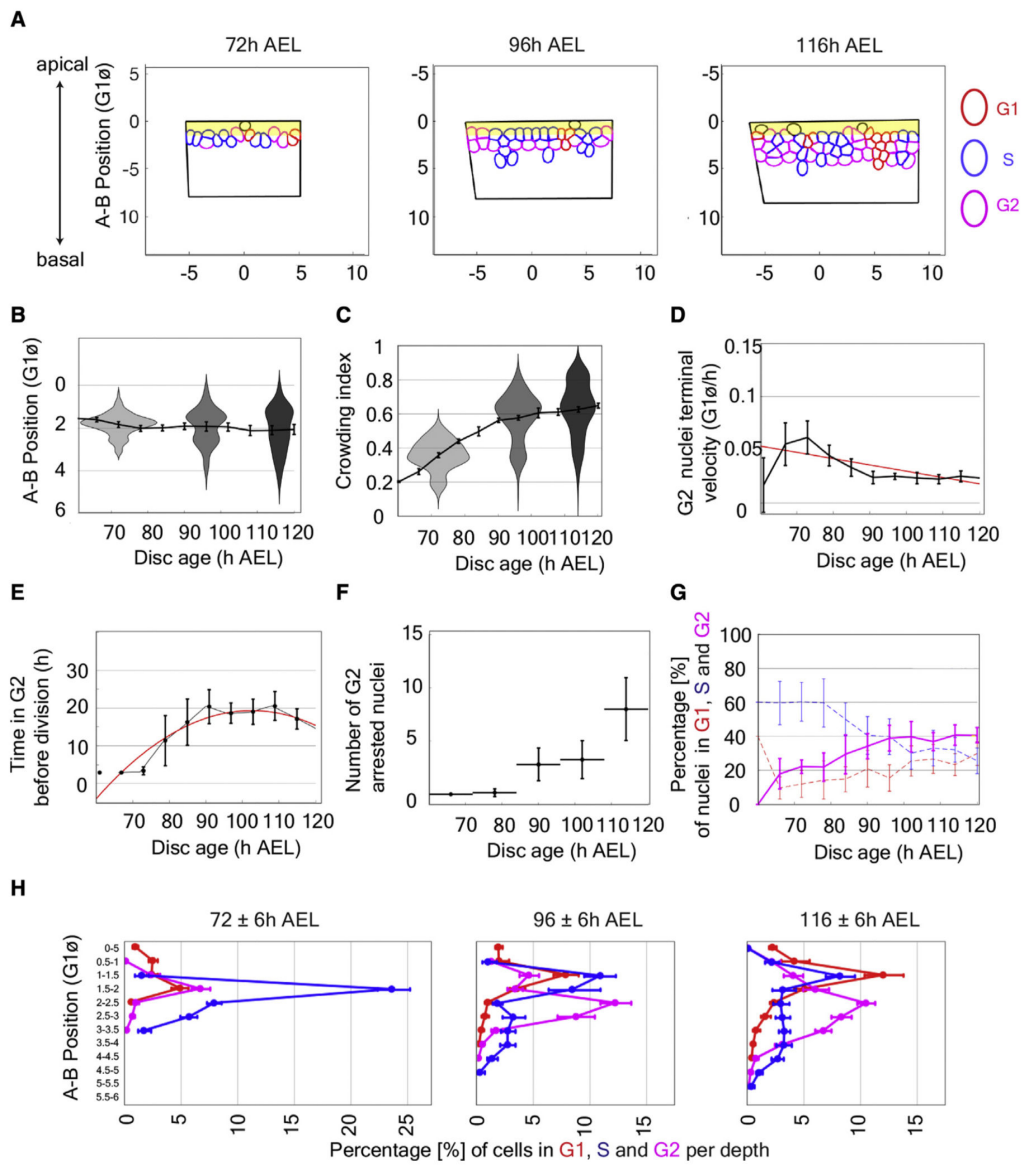
*In silico* crowding perturbs interkinetic nuclear migration and affects the distribution off cell-cycle phases (A) Snapshots of a simulation at 72, 96, and 116 h AEL. Nuclei were colored according to the cell-cycle phase: G1 is in red, S is in blue, and G2 is in magenta. A yellow ribbon represents the mitotic zone, where non-mitotic nuclei are excluded. (B) Distribution of nuclei along the apical-basal axis (expressed in units of a spherical G1 nucleus diameter, G1∅). (C) Distribution of computed crowding indices at different ages for 20 simulations. With time, nuclei occupy deeper positions, form more layers, and become increasingly crowded. (D) Terminal velocity (G1∅/h) of migrating G1 nuclei 1 h before division. This decreases as the disc “ages.” The red curve represents a polynomial. (E) Temporal evolution of G2 duration extracted from 20 simulations; a 6.5-fold increase is seen. After 90 h, G2 duration plateaued to a value of 19 h, correlating with an increase in the cumulative number of G2 nuclei that never exit G2 (“G2 arrested”). (F) Cumulative number of G2-arrested nuclei (binned in 12 h intervals). (G) Percentage of nuclei in G1, S, and G2 (averaged from 20 simulations). The proportion of G1 nuclei increases at the expense of that of nuclei in S. (H) Distribution of nuclei in G1, S, and G2 along the apical-basal axis at 12 h intervals (±6 h). Nuclei were binned in slices of ½ G1∅, and the number of nuclei in each bin was normalized to the total number of nuclei. See also Figure S2 and Video S1.

To initiate simulations with the above assumptions, the box was seeded with 10 nuclei, seven in G1 (red), three in S (blue), and none in G2 (magenta), in accordance with ratios measured in young imaginal discs.^42^ Snapshots at different times (Figures 2A and S2A, see full simulation in Video S1) suggest that, as time progresses, the number of nuclear layers, the thickness of the region occupied by nuclei, and nuclear crowding increase. This was confirmed by quantifying the output of 20 simulations, as illustrated in Figures 2B, 2C, S2B, and S2C (see details in STAR Methods). Therefore, our simulations recapitulate the key features of nuclear morphology and organization observed in fixed imaginal discs, providing support for the basic tenets of our model and allowing us to make predictions about nuclear behavior during proliferation.

One prediction of the model is that the apical-ward motion of G2 nuclei would slow down as the environment becomes increasingly crowded. Indeed, we found that the motion of virtual G2 nuclei during the hour preceding mitosis was on average 1.5-fold slower at the end of simulations than at the beginning (Figures 2D and S2D). As a consequence, G2 nuclei are predicted to need an increasing amount of time to reach the apical surface and being allowed to undergo mitosis (Figures 2E and S2E). Our simulations also predict that, with “tissue age,” an increasing number of G2 nuclei may not reach the apical surface within the duration of the simulation (Figures 2F and S2F), thus being unable to complete the cell cycle. As G2 lengthens, the proportion of G2 nuclei is expected to rise. Indeed, our simulations compute this parameter to be 17.9% at the beginning and 40.7% at the end. Interestingly, this increase was accompanied with a reduction in the computed proportion of S phase nuclei (Figures 2G and S2G) and a slowing down of the growth rate. The model also predicts a change in the spatial distribution of G2 nuclei, with a progressive accumulation in the middle of the A-B axis as the simulations progress (Figures 2H and S2H). In summary, our simulations make predictions about the rate of apical-ward movement of G2 nuclei, the duration of the G2 phase, the percentage of G2 and S nuclei, and the spatial distribution of G2 nuclei.

### Comparing the distribution of cell-cycle stages *in vivo* and *in silico*

**Figure 3:**
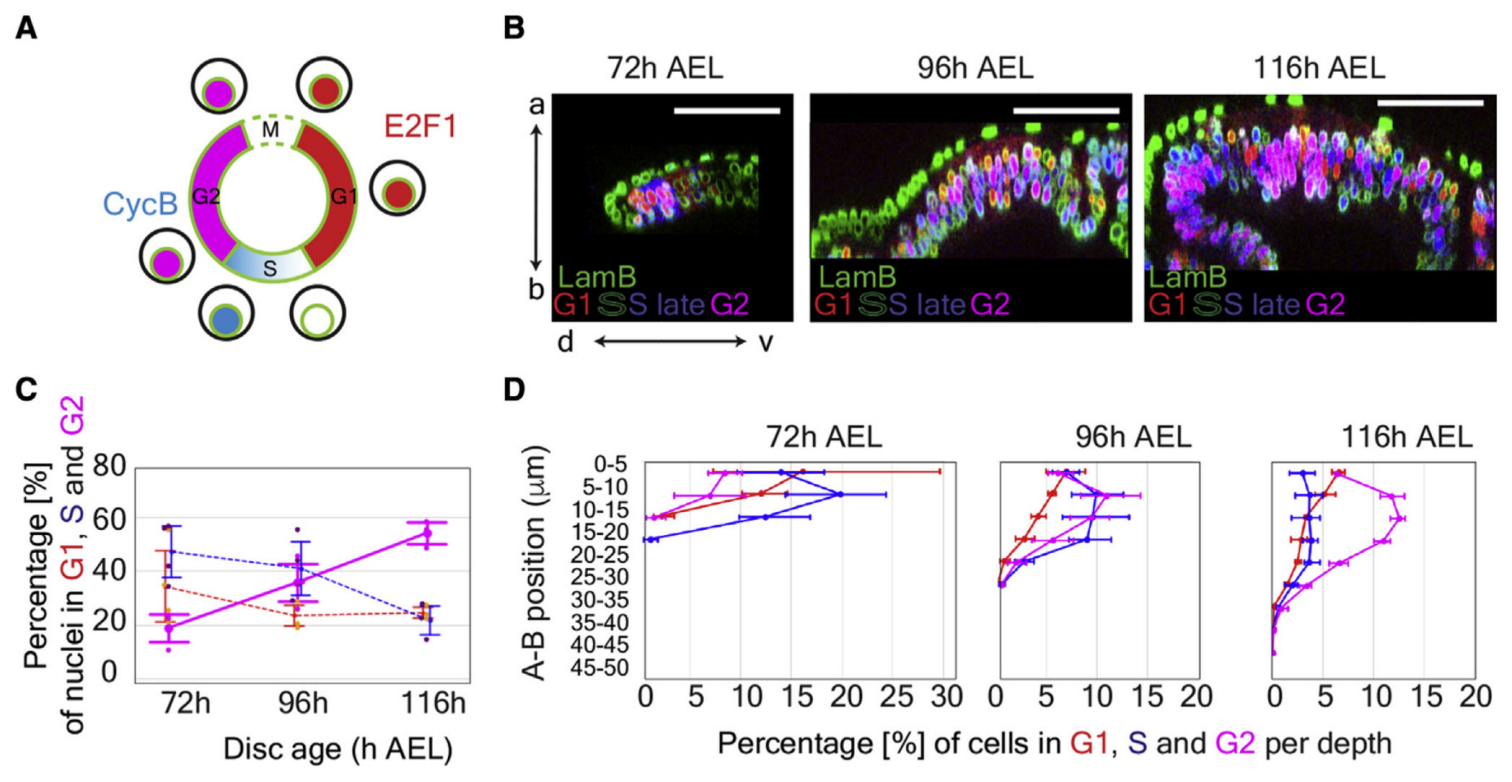
Spatiotemporal distribution of cell-cycle phases in *Drosophila* wing imaginal discs (A) Schematic representation of the FUCCI system coupled with a lamin B staining. Nuclei in G1, late S, and G2 appear in red, blue, and magenta, respectively. There is no FUCCI staining in early S, while lamin B is not detectable at M. In the following analysis, early and late S nuclei were pooled together. (B) Optical cross section of 72, 96, and 116 h AEL wing discs expressing E2F1-RFP and CycB-GFP (same preparation as those shown in Figure 1). (C) Percentage of nuclei in G1, S, and G2 at different stages. Note the increase of G2 nuclei and decrease of S nuclei, as predicted by the model. The same discs as those used to generate Figures 1A, 1B, and S1 were used (72 h AEL: 4 discs, 836 nuclei. 96 h AEL: 4 discs, 2,562 nuclei. 116 h AEL: 3 discs: 5,889 nuclei). Error bars represent standard deviation. (D) Distribution of nuclei along the apical-basal axis (μm). Nuclei in G1, S, and G2 were counted in slices of 5 μm (half the average spherical diameter of G1 nuclei) and normalized over the total number of nuclei. The relative increase of G2 nuclei at late stages is consistent with “congestion” impairing apical-ward movement. Each dot is an average from 4 discs (72 and 96 h AEL) or 3 discs (116 h AEL). (C) and (D) were generated from the same dataset. Error bars represent standard deviation. Scale bars represent 50 μm. See also Figure S3.

We now evaluate to what extent the predictions of our model are borne out by *in vivo* observations. The apical-ward velocity of G2 nuclei during IKNM was experimentally measured recently and found to decrease with age.^16^ A second prediction of our model is that the increasing duration of G2 during imaginal disc growth was inferred from the measurements of EdU (5-Ethynyl-2’-deoxyuridine) incorporation at different stages (compare Figures 2E and 2F with Figure S6D in Wartlick et al.^42^ and with Figure 2D in Neufeld et al.^43^). To assess the remaining two predictions, we used FUCCI, which allows determination of cell-cycle phases^26^ (Figure 3A). A FUCCI-encoding transgene was included in the imaginal discs used for the earlier morphometric analysis, as illustrated in Figures 3B and S3A. The proportion of nuclei in G2 was found to increase from 19.2% at 72 h AEL to 53.2% at 116 h AEL (Figures 3C and S3B). During the same period, the proportion of nuclei in S decreased 2.1-fold, while that of G1 nuclei remained constant at about 24.9% of the total number. These observations match qualitatively with the prediction of the model. We then turned to the distribution of cell-cycle phases along the A-B axis (Figures 3D and S3C). To this end, we divided the tissue along the A-B axis in 5-μm deep bins and counted the proportion of the three cell-cycle phases for all the nuclei within each bin. As expected from the fact that mitosis takes place only at the apical surface, there was an excess of G1 nuclei and a dearth of G2 nuclei in the most apical bins (both 96 and 116 h). The overall increase in the proportion of G2 nuclei was particularly noticeable in the middle of the A-B axis, in accordance with our simulations. In the simulations, the A-B distributions of G2 and G1 nuclei did not match, as they do *in vivo*. Nevertheless, the simulations qualitatively recapitulated several *in vivo* observations, including the increases in nuclear layers and crowding, the changes in proportions of nuclei in the different cell-cycle phases, the lengthening of the G2 phase duration, and the reduction in the terminal G2 speed.

### A basal signal could impose a second gate to cell-cycle progression

**Figure 4:**
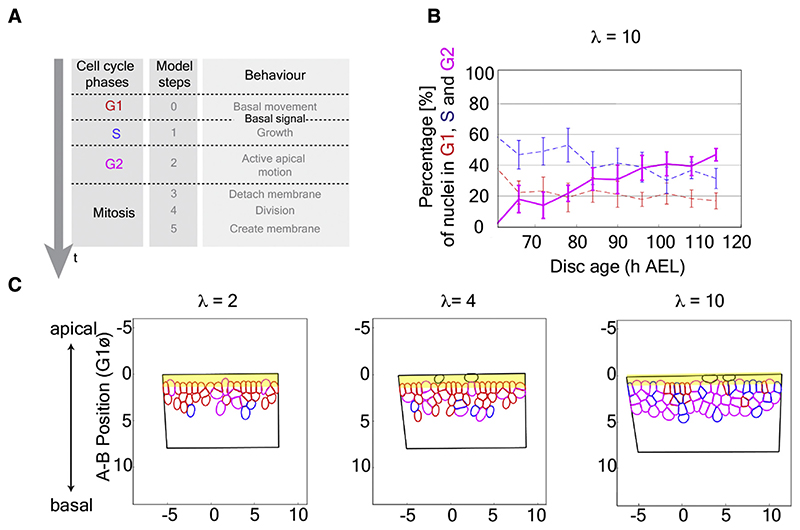
A two-gate model of IKNM: a basal signal could regulate nuclear layering, crowding, and proliferation rates (A) Modification of the model to include a hypothetical basal signal that triggers the G1-S transition. Only the duration of S is defined a priori, whereas the durations of G1 and G2 are outputs. The range of this signal (*λ*) was expressed in multiples of G1 nuclear diameters (see Sup. Exp. Pro. Annex 1 for more details). (B) Simulations with *λ* = 10 nuclear diameters recapitulated the increase of G2 percentage that occurs as the tissue grows. (C) Snapshots of simulation output at 116 h AEL with *λ* = 2, *λ* = 4, or *λ* = 10. G1 nuclei are colored in red, S in blue, and G2 in magenta. A yellow ribbon represents the mitotic zone where non-mitotic nuclei are excluded. See also Figure S4 and Videos S2, S3, and S4.

According to our model, nuclei progressively undergo cell-cycle arrest as they become increasingly unable to reach the apical surface. However, if apical localization was the only gate to cell-cycle progression, apical nuclei would be expected to proliferate indefinitely. Since this is not observed *in vivo*, we hypothesize that an additional signal controls cell-cycle progression. For example, one could envision that a basal signal is required for S phase entry, forcing nuclei to move basally if they are to continue cycling. Although hypothetical, the existence of a basal signal is not without precedent since basal Wnt5 has recently been shown to control IKNM in the small intestine of the mouse.^44^ Moreover, since the basal surface of wing imaginal discs is facing the circulation, a basal signal could mediate systemic control of cell-cycle progression, allowing tissue intrinsic and extrinsic influences to be integrated. We formalized the requirement for a basal signal by modifying assumption A4 (Figure 4A; Methods S1). In this framework, the duration of G1 no longer needs to be specified a priori. Nevertheless, the model was still able to recapitulate all the experimentally observed features, including the proportion of cell-cycle phases observed over time *in vivo* (Figures 4B and S4A–S4E). In addition, the refined model confirmed the expectation that increasing the range of the basal signal would lead to a larger number of nuclear layers (Figures 4C and S4F; Videos S2, S3, and S4), perhaps by allowing nuclei to enter the S phase more rapidly (Figure S4G).

## Conclusions

Here, we have taken a computational approach to investigate how mechanical constraints could impact on IKNM and hence proliferation in a pseudostratified epithelium. Previous models of nuclear mechanics within tissues have either taken a macroscopic view^38,45,46^ or have considered a microscopic view without allowing nuclear deformation.^24,47^ By representing nuclei as 20-sided polygons, we were able to infer their deformability, compute the forces that impact their movement, and thus build a mechanical model of IKNM. Our model was able to reproduce experimentally observed features of growing wing imaginal discs, including progressive nuclear layering, the distribution of cell-cycle phases across the A-B axis, and the accumulation of G2 nuclei with time. It also confirmed earlier suggestions that “congestion,^48^” “traffic bottleneck,^23^” or nuclear density^16^ affect the apical-ward component of IKNM. Crowding is also expected to impede basal-ward movement, which is needed to make space for incoming G2 nuclei and also possibly to allow G1 nuclei to access a basal signal needed for S phase entry. Such a signal remains hypothetical, but the need for nuclei to sample both the apical and basal regions for cell-cycle progression would explain why IKNM is such a common feature of developing epithelia.^25,30^ Our study adds nuclear crowding to the list of processes that could contribute to growth deceleration in developing tissues, besides nutrient access, dwindling growth factor signaling, changes in hormonal control,^2,9,49^ and/or mechanical feedback through adherens junctions. It remains a challenge to figure out how these processes are genetically controlled and integrated to ensure reproducible tissue size in a wide variety of conditions.

## Star Methods

### Key Resources Table

**Table:** 

REAGENT or RESOURCE	SOURCE	IDENTIFIER
Antibodies		
anti-Lamin B	DSHB	ADL67.10-s
anti-Lamin B	DSHB	ADL84.12-s
anti-mouse Alexa Fluor Plus 647	Invitrogen	A32728
Critical commercial assays		
FocusClear™	2Bscientific	FC-101
MountClear™	2Bscientific	MC-301
Experimental models: Organisms/strains		
*D. Melanogaster UAS-FUCCI*	Bloomington	55101
*D. Melanogaster pdm2^R11F02^-Gal4*	Bloomington	49828
*D. Melanogaster nub*-Gal4	Bloomington	86108
D. Melanogaster *tubulin-Gal4*	Bloomington	5138
D. Melanogaster *UAS-CD8-GFP*	Bloomington	5130
Software and algorithms		
Fiji	Schindelin et al.^50^	RRID: SCR_002285
Nessys	Blin et al.^51^	https://pickcellslab.frama.io/docs/use/features/segmentation/nessys/
ActiveUnetSegmentation	Smith^52^	N/A
MATLAB R2016b & R2020b	MathWorks	RRID: SCR_001622
Scipy	https://scipy.org/	RRID: SCR_008058
Numpy	https://numpy.org/	RRID: SCR_008633
Scikit-image	https://scikit-image.org/	RRID: SCR_021142
Seaborn	https://seaborn.pydata.org/	RRID: SCR_018132
Code for mathematical simulations	This work	https://doi.org/10.5281/zenodo.6190050

### Resource Availability

#### Lead contact

Further information and requests for resources and reagents should be directed to and will be fulfilled by the lead contact, Jean-Paul Vincent (jp.vincent@crick.ac.uk).

#### Materials availability

This study did not generate new unique reagents.

#### Data and code availability

- All data reported in this paper will be shared by the lead contact upon request.
- All original code has been deposited at zenodo.org/record/6190050#.YhPPxi2ZPdR and is publicly available as of the date of the publication. DOIS are listed in the key resource table.
- Any additional information required to reanalyse the data reported in this work paper is available from the lead contact upon request.

### Experimental Model and Subject Detail

Flies were reared in standard cornmeal/agar media at 25C. Larvae were staged from the time of L2-L3 transition. The following strains were obtained from the Bloomington stock center:

nubbin-Gal4, UAS-FUCCI (UAS-GFP.E2f1.1-230, UAS-mRFP1.NLS.CycB.1-266 on the III) and pdm2R11F02-Gal4.

### Methods Details

#### Genotypes

Figures 1A, 1B, 3B–3D, S1, and S3: the same dataset of 11 discs was used in these figure panels. For the 96h and 116h AEL wing discs, the genotype was *nubbin-Gal4/UAS-FUCCI* and for the 72h AEL wing discs, it was *pdm2^R11F02^-Gal4/UAS-FUCCI. Pdm2^R11F02^*-Gal4 is a pouch marker which is stronger at 72h AEL than *nubbin-gal4.^53^*

Figure S1B: *tub-G4/UAS-CD8-GFP*

#### Immunohistochemistry and imaging

Wing imaginal discs were fixed in 4% formaldehyde for 40 min using standard procedures. The discs were then incubated overnight at 4°C with a mixture of two anti-Lamin B (1:100, ADL67.10-s, DSHB and ADL84.12-s in PBS with TritonX at 0.5%) antibodies, followed by two hours in anti-mouse Alexa Fluor Plus 647 (1:1000, A32728, Invitrogen) at room temperature. To preserve 3D structure, the fixed and stained discs were deposited in warm low melting agar (1% low melting point agar (A9414 Sigma- Aldrich) in PBS). 10μl of liquid agar containing the wing disc was then transferred onto a 1.5x coverslip. Before solidification of the agar, the wing disc was positioned at the bottom of the drop, with the pouch area facing down. The drop was surrounded with a ring of silicone grease (Z273544 Aldrich), creating a small chamber. 10μl of FocusClear™ (FC-101, 2Bscientific) was then added on top of the agar drop and allowed to act for 1h in a dark humid chamber. Subsequently, FocusClear™ was removed and 20μl of MountClear™ (MC-301,2Bscientific) was added. A slide was then positioned on top of the grease to close the chamber. The slide was then inverted, and the discs were imaged with an upright Leica SP5 confocal microscope equipped with a 63x glycerol (1.3 NA) objective, with a pixel size of 0.24 × 0.24 μm and a z step of 0.7 μm.

#### Image analysis

Before segmentation, the region of interest was manually cropped using FIJI.^50^ For eight of the eleven discs analysed, nuclei were segmented using the Nessys module of PickCells.^51^ For the 3 remaining discs a machine learning algorithm (see below) was used to generate a binary mask of the segmented nuclei. This binary mask was then fed into Nessys to segment individual nuclei.

Nessys then calculated the center of mass, volume, length of the longest axis (l_max_) as well as the mean fluorescence values in the different channels (E2F1 and CycB) inside each segmented nuclei.

To rotate the sample, a custom-made python code using the Numpy and Scikit-image libraries was used to define a plane based on three points manually picked and located in the most apical part of the disc. This plane coupled to a normal vector allowed to define a new frame-of-reference, and to re-calculate the coordinates of each of the center of mass of the nuclei.

The wing disc curvature was accounted for by first binning the nuclei in squares defined orthogonally to the apical plane. The coordinates of the most apical nucleus were then used as the reference point to recalculate the position along the z-axis (depth) of all the other nuclei present in the bin.

The cell-cycle phase was determined by comparing the binarized values of the E2F1 and CycB signals (Figure 3A). Nuclei in early or late S phase were pooled together in all the analyses and considered as S phase.

Crowding was calculated by first generating a 3D box surrounding each nuclei. This box was 30 pixels bigger than the most extreme values of the nucleus on the x and y axis and 10 pixels on the z axis. Then, after ignoring the voxels containing the nucleus of interest for the analysis, for each box, the number of voxels containing another nucleus (volume of surrounding nuclei) was divided by the total number of voxels (theoretical available volume). Nuclei located at a distance below 4 μm of the border of the segmentation area where ignored.

#### Machine learning for the segmentation of the nuclei

Images were processed using a modified 3D Unet^54^ to create a distance transform that Nessys^51^ could segment. The network produced 3 output layers: the nuclei’s boundary, a mask of nuclei and background, and a distance transform of segmented images. Training labels were created using segmentation outputs from Nessys. Each labelled cell was converted into a binary mask, a binary border, and a distance transform. The distance transform was performed by eroding the binary blob that represents an individual cell. Our unet implementation using python and tensorflow source code is available online.^52^

#### Mathematical simulations

Simulations were performed according to the model and method described in Methods S1.

### Quantification and Statistical Analysis

Data for the sample number (number of wing discs, nuclei or simulations), statistical significance (represented as* p<0.05, ** p<0.01, *** p<0.001) as well as dispersion measures (standard deviation) is given in the figures and the figures legends. All statistical tests were performed using the stats module from the SciPy python library.

A Wilcoxon signed rank sum test was used after testing for normality using a Shapiro-Wilk test. There was no blinding performed.

The graphs in Figures 1B, 2H, 3C, S1D, S1F–S2H, S3B, S3C, and S4E were performed using the python libraries Seaborn and MatplotLib. The graphs in Figures 2B–2G, 4B, S2B–S2G, S4A–S4D, and S4F–S4M were performed with MATLAB.

## Supplementary Material

Figure S1

Methods S1

Video S1

Video S2

Video S3

Video S4

## Highlights

- As *Drosophila* wing imaginai discs grow, nuclei become increasingly crowded
- An agent-based model predicts that nuclear crowding impairs nuclear movement
- The model also recapitulates the distribution of cell-cycle phases during growth
- Interkinetic nuclear migration could allow nuclei to access cell-cycle signals

## In brief

Hecht et al. combine mathematical modeling with morphometric analysis to suggest that as Drosophila wing imaginal discs grow, nuclear crowding increases, slowing down interkinetic nuclear migration. This in turn could affect cellcycle progression by impairing the ability of nuclei to access signals required for S phase entry and mitosis.
