# Supplementary material for: Mechanical constraints to cell-cycle progression in a pseudostratified epithelium
Source: Curr Biol. Author manuscript; Available in PMC 2023 Sep 7. (PMC7615048; doi:10.1016/j.cub.2022.03.004)
Supplement: Figure S1 [file EMS186829-supplement-Figure_S1.pdf]

Current Biology, Volume *32*

Supplemental Information

Mechanical constraints to cell-cycle progression

in a pseudostratified epithelium

Sophie Hecht, Gantas Perez-Mockus, Dominik Schienstock, Carles Recasens-Alvarez, Sara Merino-Aceituno, Matthew B. Smith, Guillaume Salbreux, Pierre Degond, and Jean-Paul Vincent

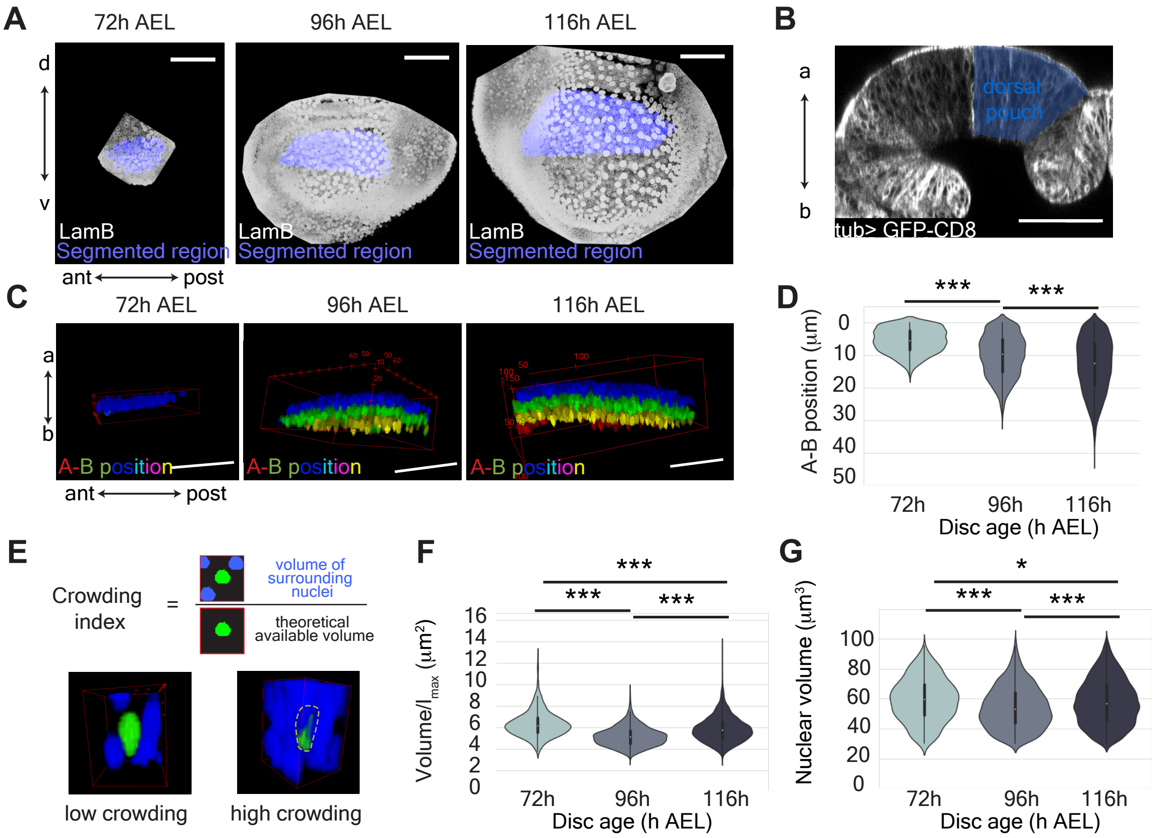
A
72h AEL
96h AEL
116h AEL
d
v
LamB
Segmented region
ant
post
B
a
b
dorsal
pouch
tub> GFP-CD8
C
72h AEL
96h AEL
116h AEL
a
b
A-B position
ant
post
D
***
***
A-B position (µm)
0
10
20
30
40
50
72h
96h
116h
Disc age (h AEL)
E
Crowding
index
=
volume of
surrounding
nuclei
theoretical
available volume
low crowding
high crowding
F
***
***
***
Volume/I$_{max}$ (µm^2)
16
14
12
10
8
6
4
2
0
72h
96h
116h
Disc age (h AEL)
G
*
***
***
Nuclear volume (µm^3)
100
80
60
40
20
0
72h
96h
116h
Disc age (h AEL)

Figure S1: Nuclear organisation in a growing wing imaginal disc of Drosophila. Related to Figure 1. (A) Max projection of 72h, 96h and 116h AEL wing imaginal discs stained with anti-Lamin B (white) and mounted in an agar drop infused with FocusClear^{TM}. The Nessys Module of the PickCells program, and custom algorithms (see Methods) were used to identify nuclei. Our analysis was confined to the pouch, the area that gives rise to the wing proper. And to ease segmentation, which still requires manual correction, only the dorsal compartment was considered since its behaviour can be considered representative of the whole pouch ([S1,S2]). Nuclei located near the dorso-ventral boundary were not included in the analysis since they terminate proliferation in response to a specific genetic program [S3]. Thus, in the blue-shaded areas, we catalogued 836 nuclei from 4 discs at 72h after egg laying (AEL), 2562 nuclei from 4 discs at 96h AEL and 5889 nuclei from 3 wing discs at 120h AEL. **(B)** Optical cross-section of a 116h AEL wing imaginal disc expressing CD8-GFP to highlight cell membranes. The region where nuclei were segmented (the dorsal compartment) is shaded blue. (**C)** 3D reconstruction of segmented nuclei from the discs showed in A and C. Nuclei are colour-coded according to their depth along the apical-basal axis. Note the increased number of layers. (**D)** Distribution of nuclei along the apical-basal (in μm) axis in 72h, 96h and 116h AEL wing discs. As discs grow, nuclei are increasingly occupying more basal positions (72h AEL: 4 discs, 836 nuclei. 96h AEL: 4 discs, 2562 nuclei. 116h AEL: 3 discs: 5889 nuclei). **(E)** Crowding index and examples of a low and high crowding situation. A crowding index was measured for each nucleus by first creating a box enclosing the nucleus of interest 30 pixels beyond the edge of the nuclei in the x and y axis, and 10 pixels in the z axis. We then measured the proportion of voxel occupied by other nuclei (volume of surrounding nuclei) in this box (theoretical available volume). **(F-G)** Nuclear 'roundness' (V/l^{max}) along the A-B axis and the distribution of nuclear volumes (μm^3) at three developmental times. The biphasic trend for both parameters (down between 72 and 96 hAEL and up between 96 and 116 hAEL) is consistent with the observations of Kirkland and colleagues [S4]. This is not predicted by the model; it could result from additional physiological or mechanical features not incorporated in our model [S5-S7]. Data was from 72h AEL: 4 discs, 836 nuclei. 96h AEL: 4 discs, 2562 nuclei. 116h AEL: 3 discs: 5889 nuclei. Wilcoxon rank-sum statistic test for two samples was performed in B-D. * P<0.5 *** P<0.001. The scale bars in the figure represents 50 μm.

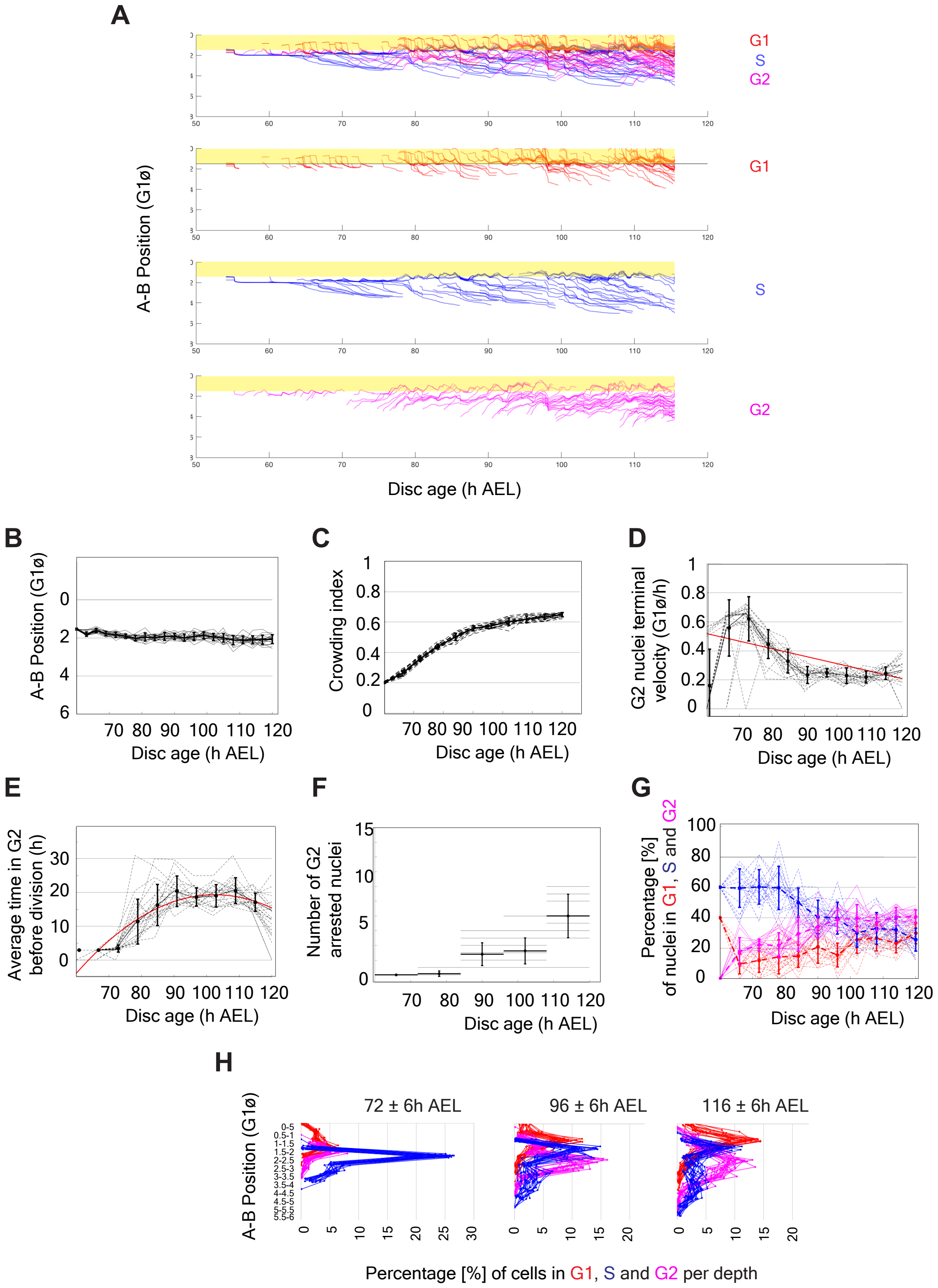
A
G1
S
G2
A-B Position (G1ø)
Disc age (h AEL)
B
A-B Position (G1ø)
Disc age (h AEL)
C
Crowding index
Disc age (h AEL)
D
G2 nuclei terminal velocity (G1ø/h)
Disc age (h AEL)
E
Average time in G2 before division (h)
Disc age (h AEL)
F
Number of G2 arrested nuclei
Disc age (h AEL)
G
Percentage [%] of nuclei in G1, S and G2
Disc age (h AEL)
H
72 ± 6h AEL
96 ± 6h AEL
116 ± 6h AEL
A-B Position (G1ø)
Percentage [%] of cells in G1, S and G2 per depth

Figure S2: Cell cycle phase analysis in simulated epithelia. Related to Figure 2. (A) Apical-basal position (G1∅) of individual nuclei G1 (red), S (blue) and G2 (magenta) over the course of a representative simulation. The yellow ribbon represents the mitotic zone where non-mitotic nuclei are excluded. Panels B-H show parameters for each of the 20 simulations performed: **(B)** average apical-basal position (G1∅) **(C)** average crowding index, **(D)** average terminal G2 velocity (G1∅/h) **(E)** average time spent in G2 before mitosis (h), **(F)** number of G2 arrested nuclei. **(G)** Percentage of nuclei in the different phases along time, and **(H)** nuclear distribution along apical-basal axis (G1∅), at 72 +- 6h, 96 +- 6h, and 116 +- 6h.

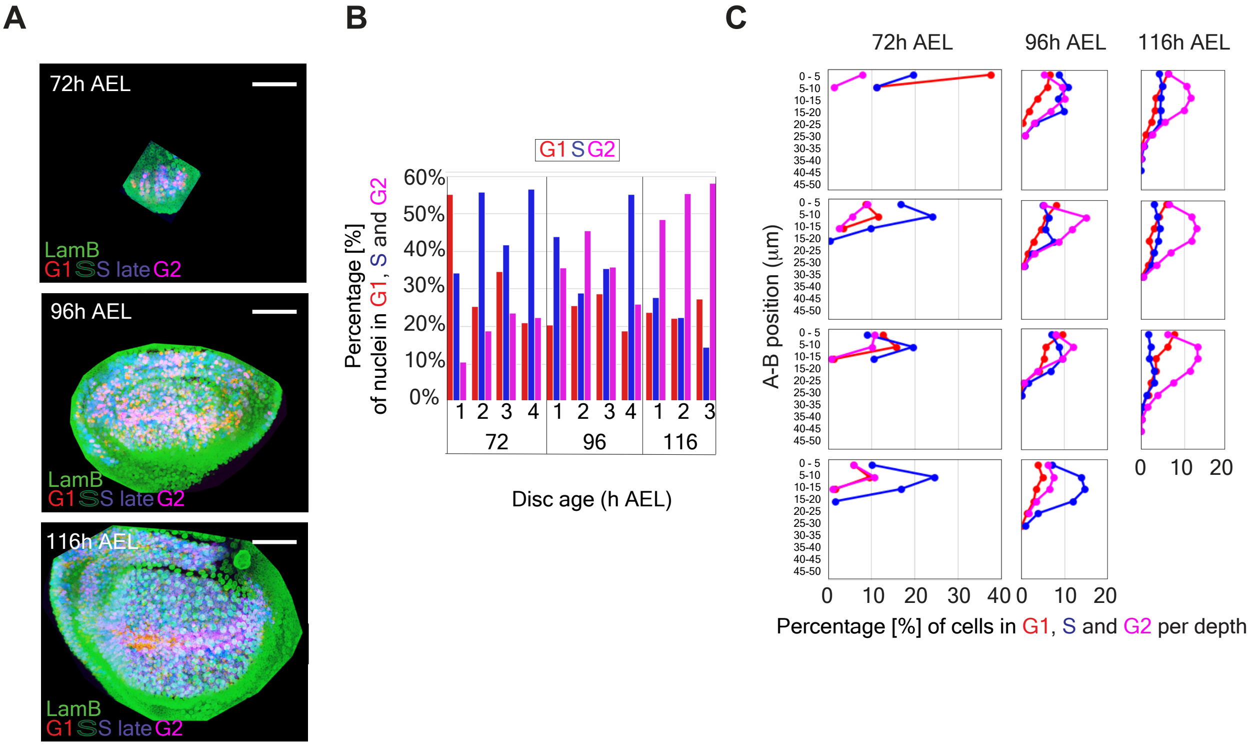
A
B
C
72h AEL
96h AEL
116h AEL
LamB
G1 S S late G2
G1 S G2
Percentage [%] of nuclei in G1, S and G2
60%
50%
40%
30%
20%
10%
0%
1 2 3 4 1 2 3 4 1 2 3
72
96
116
Disc age (h AEL)
A-B position (µm)
0 - 5
5-10
10-15
15-20
20-25
25-30
30-35
35-40
40-45
45-50
0 10 20 30 40
0 10 20
Percentage [%] of cells in G1, S and G2 per depth

Figure S3: Spatio-temporal distribution of cell cycle phases in Drosophila wing imaginal discs. Related to Figure 3. (A) Max projection of wing imaginal discs expressing FUCCI (multiple colors) stained with anti-Lamin (Green). Nuclei in G1 appear in red, S appear either unmarked with FUCCI (still stained with the nuclear marker) or blue, and G2 appear in magenta. **(B)** Proportion of nuclei in the different phases for every disc analysed. 72h_disc1: 152 nuclei. 72h_disc2: 233 nuclei. 72h_disc3: 285 nuclei. 72h_disc4: 166 nuclei. 96h_disc1: 417 nuclei. 96h_disc2: 926 nuclei. 96h_disc3: 635 nuclei. 96h_disc4: 584 nuclei. 116h_disc1: 1892 nuclei. 116h_disc2: 1663 nuclei. 116h_disc3: 2334 nuclei. **(C)** Distribution of nuclei along apical-basal axis (μm) for every imaginal disc analysed.

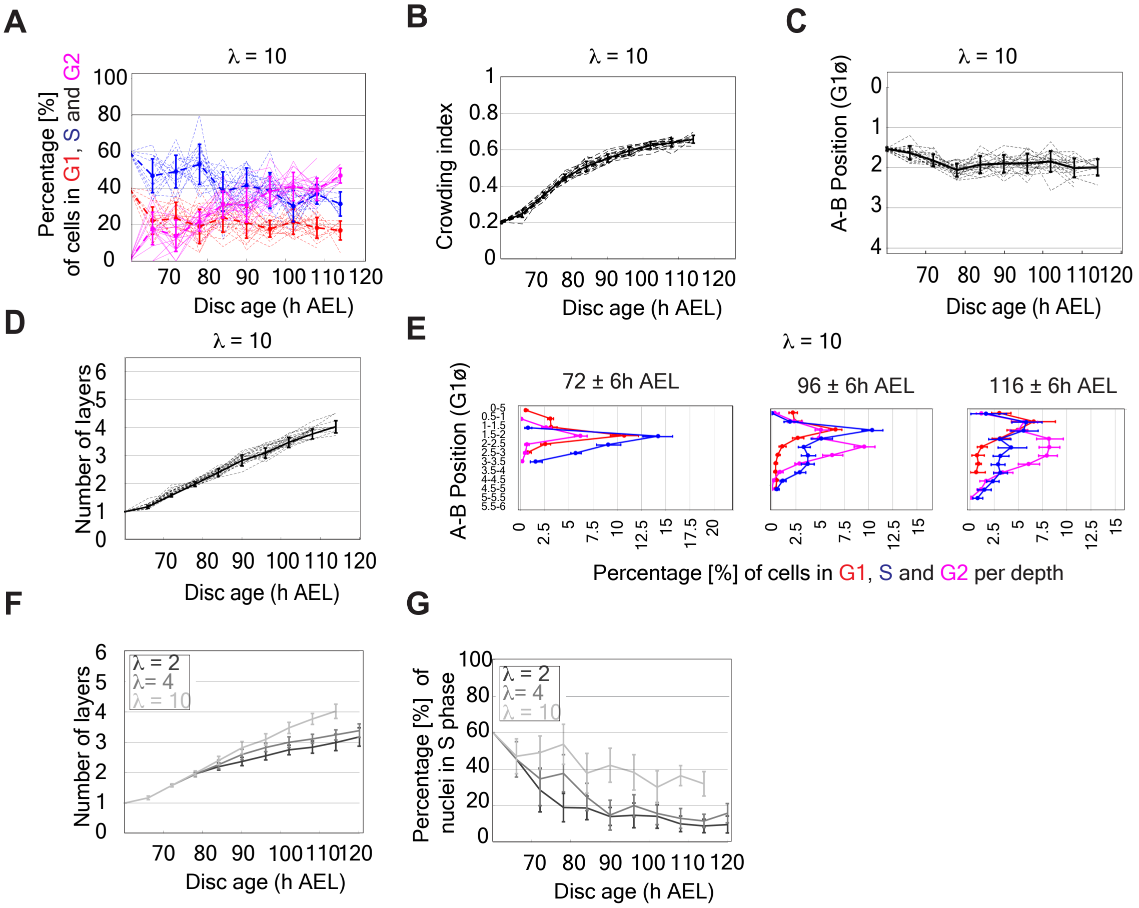

A
λ = 10
Percentage [%] of cells in G1, S and G2
Disc age (h AEL)
B
λ = 10
Crowding index
Disc age (h AEL)
C
λ = 10
A-B Position (G1ø)
Disc age (h AEL)
D
λ = 10
Number of layers
Disc age (h AEL)
E
λ = 10
72 ± 6h AEL
96 ± 6h AEL
116 ± 6h AEL
A-B Position (G1ø)
Percentage [%] of cells in G1, S and G2 per depth
F
λ = 2
λ= 4
λ = 10
Number of layers
Disc age (h AEL)
G
λ = 2
λ= 4
λ = 10
Percentage [%] of nuclei in S phase
Disc age (h AEL)

Figure S4: Computed spatio-temporal distribution of cell cycle phases and other parameters (multiple simulations). Related to Figure 4. For panels A-E, λ = 10, while panels F-G explore the effect of λ on nuclear behaviour. **(A)** Percentage of cells in the different phases of the cell cycle over time. **(B)** Average crowding index. **(C)** Average apical-basal position (G1$\emptyset$). **(D)** Number of nuclear layers. **(E)** Distribution of cell cycle phases along the apical-basal axis (G1$\emptyset$) at 72 +- 6h, 96 +- 6h and 116+- 6h AEL. **(F-G)** Increasing the range of the basal signal leads to an increased in the number of nuclear layers and a higher rate of proliferation (quantified by the percentage of nuclei in the S phase). The average of 20 simulations is represented in panels B, C, E, F and G. Error bars represent standard deviations.

SUPPLEMENTAL REFERENCES

S1. Dye, N.A., Popovic, M., Iyer, K.V., Fuhrmann, J.F., Piscitello-Gomez, R., Eaton, S., and Julicher, F. (2021). Self-organized patterning of cell morphology via mechanosensitive feedback. Elife *10*. 10.7554/eLife.57964.

S2. Worley, M.I., Setiawan, L., and Hariharan, I.K. (2013). TIE-DYE: a combinatorial marking system to visualize and genetically manipulate clones during development in Drosophila melanogaster. Development *140*, 3275-3284. 10.1242/dev.096057.

S3. Neufeld, T.P., de la Cruz, A.F., Johnston, L.A., and Edgar, B.A. (1998). Coordination of growth and cell division in the Drosophila wing. Cell *93*, 1183-1193. 10.1016/s0092-8674(00)81462-2.

S4. Kirkland, N.J., Yuen, A.C., Tozluoglu, M., Hui, N., Paluch, E.K., and Mao, Y. (2020). Tissue Mechanics Regulate Mitotic Nuclear Dynamics during Epithelial Development. Curr Biol *30*, 2419-2432 e2414. 10.1016/j.cub.2020.04.041.

S5. Mirth, C.K., and Shingleton, A.W. (2012). Integrating body and organ size in Drosophila: recent advances and outstanding problems. Front Endocrinol (Lausanne) *3*, 49. 10.3389/fendo.2012.00049.

S6. Tozluoglu, M., Duda, M., Kirkland, N.J., Barrientos, R., Burden, J.J., Munoz, J.J., and Mao, Y. (2019). Planar Differential Growth Rates Initiate Precise Fold Positions in Complex Epithelia. Dev Cell *51*, 299-312 e294. 10.1016/j.devcel.2019.09.009.

S7. Mirth, C.K., Truman, J.W., and Riddiford, L.M. (2009). The ecdysone receptor controls the post-critical weight switch to nutrition-independent differentiation in Drosophila wing imaginal discs. Development *136*, 2345-2353. 10.1242/dev.032672.
