# Supplementary material for: Mechanical constraints to cell-cycle progression in a pseudostratified epithelium
Source: Curr Biol. Author manuscript; Available in PMC 2023 Sep 7. (PMC7615048; doi:10.1016/j.cub.2022.03.004)
Supplement: Methods S1 [file EMS186829-supplement-Methods_S1.pdf]

METHODS S1: NUMERICAL SIMULATIONS

In this section we describe the Individual-Based Models used for simulating the imaginal disc of Drosophila. The flowchart presented in Fig. A1 explains the structure of the model. The model is constructed such that at each time step the variables of the system are at a minimal energy state. Then at each time step the system is disrupted by biological phenomena. These phenomena are linked to the growth of the tissue and to the evolution of the nuclei, including their growth and motion during IKNM. The minimal energy state is then restored by minimising the energy functional subject to constraints. Therefore, the model was developed in an optimisation framework.

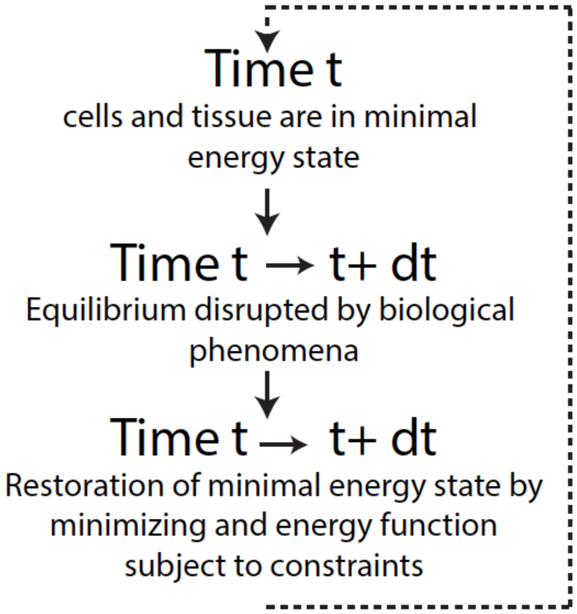

Fig. A1: Flowchart of the Individual Based Model.

We first introduce the different agents of the model, as well as the energies and constraints inherent to the biological relevance of the model with no consideration of time. Secondly, the minimisation problem is introduced, including the minimisation algorithms and the choice of the minimisation parameters. Then, a description of the time dependent part of the model is provided. Last, we present the choice of parameters.

Choice of the agents

Because the aim of the model is to study the influence of crowding on IKNM during tissue growth, the nuclei are a key component of the model. We chose to develop an off-lattice model where each nucleus is represented individually by a polygon. The choice of polygons is critical because it allows the deformation of nuclei, which is known to take place[S1]. Let N be the number of cells in the tissue. For each cell $i = 1, \dots, N$, its nucleus is represented by a polygon of N_p vertices. The position of the vertices is given by $X = (X_i^k)_{i \in [1,N], k \in [1,N_p]}$ with

$$X_i^k = (x_i^k, y_i^k) \quad \forall k \in [1; N_p], \forall i \in [1; N].$$

To simplify the notations in the following sections, we introduce some quantifiers that are specific to the nuclei. These quantifiers are not variables of the model and depend entirely on the polygon vertices X representing the nuclei. For each cell i=1,...,N, we denote as follows:

- $X_i = \frac{1}{N_p}\sum_{k=1}^{N_p} X_i^k$, the position of the center of mass of the nucleus i.
- $S_i = \sum_{k=1}^{N_p}\frac{1}{2}|X_iX_i^k \wedge X_iX_i^{k_+}|$, the surface of the nucleus i. The surface S_i of each nucleus i is computed by adding the area of every triangle $X_iX_i^kX_i^{k_+}$, with $k \in [1; N_p]$ and $k_+ \equiv k+1\ [N_p]$. The wedge symbol denotes the cross product between two vectors and [.] denotes the modulo operation (i.e. k [N] is the remainder in the Euclidean division of k by N). .
- $R_i = \sqrt{\frac{S_i}{\pi}}$, the radius such that the surface S_i of the nucleus i is equal to the surface of a ball with radius R_i.

The tissue is represented by an elastic box. We denote the edges of the box by $Y = (Y_j)_{j\in\{1,2,3,4\}} \in \mathbb{R}^2$ and consider the notations $j_\pm \equiv j \pm 1\ [4]$. The vertices of the box Y_j and Y_{j_+} are linked by springs of stiffness k_j^s and rest length l_j^s for $j \in [1,4]$. In addition, the angles of the box $(Y_{j\_}Y_jY_{j_+})$ are linked by torques of stiffness k_j^t and rest angle θ_j^t for $j \in [1,4]$. The springs and torques linking the vertices of the box allow deformation of the box to accommodate possible internal pressure created by the nuclei. Since the model aims to describe nuclear motion along the apical/basal (A-B) axis during the cell cycle, it is essential that the movements of the nuclei are restricted by vertical upper and lower bounds, representing the apical and basal surfaces.

Another major component of the model is the cable that mimics the effect of the cell membrane and associated cortex, which connects the basal to the apical surfaces. While these cables are not variables of the model, they are one of its essential ingredients. Indeed, their role of constricting the lateral movement of nuclei cannot be ignored when studying the motion of the nuclei during development. To avoid adding a lot of complexity to the model, the cell cortices are represented by an abstract straight line. For each cell i with $i = 1,...,N$, the coordinates of the attachment point of the cortex to the basal and apical surfaces are given by $M_i^b = (xm_i^b, ym_i^b) \in \mathbb{R}^2$ and $M_i^a = (xm_i^a, ym_i^a) \in \mathbb{R}^2$, respectively. The positions of the apical attachment point is fixed while the positions of the basal attachment can evolve over time to accommodate the surrounding forces.

Energies

Because the agents of the model rely on springs and torques, different energies arise from the system. These energies inform us on the way the elements of the model behave. We distinguish three categories for the energies of the model:

- The tissue energy $E_{tissue} = E_{box} + E_{ap}$ composed of the energy E_{box}related to the elasticity of the box and of the apical energy E_{ap}. The assembly of spring and torques linking the vertices of the box creates the energy of the box. It is given by

$$E_{box}(Y) = \sum_{j=1}^{4}\frac{1}{2}k_j^s\left(\frac{|Y_j - Y_{j_+}| - \tau_j^s}{\tau_j^s}\right)^2 + \sum_{j=1}^{4}\frac{1}{2}\frac{k_j^t}{\tau_j^{t^2}}\left(\frac{Y_j - Y_{j_+}}{|Y_j - Y_{j_+}|}\cdot\frac{Y_j - Y_{j_-}}{|Y_j - Y_{j_-}|} - \tau_j^t\right)^2,$$

  where $j_- \equiv j - 1\ [4]$ for $j \in [1,4]$ and $(\tau_j^s)_{j\in[1,4]}$, $(\tau_j^t)_{j\in[1,4]}$ are the rest lengths and angles of the springs and torques respectively. The apical energy E_{ap} models a region

of length l^a near the apical surface where only dividing nuclei can enter. The expression of E_{ap} is calculated as follows:

$$E_{ap}(X,Y) = \sum_{i=1}^{N} \frac{1}{2} k_j^a \left(l^a - d_{(Y_1Y_2)}(X_i)\right)_+^2,$$

with the function $(u)_+ = \max(0,u)$ is the positive part function and $d_{(D)}(Z)$ is the distance of the point Z to the straight line (D).

- The nuclei energy E_{nuclei} models the cost of the deformation of the nuclei. In a resting position the nuclei are supposed to be in a spherical configuration. The nuclei energy E_{nuclei} is decomposed into two energies: the bending energy E_{bend}, acting on the angles of the polygon, and the perimeter energy E_{per}, acting on the lengths of the edges. These two energies are expressed by

$$E_{bend} = \sum_{i=1}^{N} \sum_{k=1}^{N_p} \frac{1}{2} k_1^b \left(\frac{\cos(\theta_i^k) - \cos(\theta_0^b)}{\cos(\theta_0^b)}\right)^2 + \sum_{i=1}^{N} \sum_{k=1}^{N_p} \frac{1}{2} k_1^b \left(\frac{\sin(\theta_i^k) - \sin(\theta_0^b)}{\sin(\theta_0^b)}\right)^2,$$

$$E_{per} = \sum_{i=1}^{N} \sum_{k=1}^{N_p} \frac{1}{2} k^p \left(\frac{|X_i^k X_i^{k+}| - l_0^b}{l_0^b}\right)^2,$$

where θ_i^k is the angle $X_i^{k-} X_i^k X_i^{k+}$ and k_1^b, k_2^b, k^p are the stiffness of the energies and with $\theta_0^b = \frac{2\pi}{N_p}$ and $l_0^p = \frac{2\pi R_i}{N_p}$.

- The energy $E_{cable-to-nuclei}$ represents the action of the cable representing the cell cortex on the nuclei. In the tissue, for a given cell it is clear that the cell cortex constricts the lateral movement of the nucleus. In addition, we suppose that the cortex constraints the shape of nuclei. Then we consider that each vertex of a polygon X_i^k is linked by a spring of rest length zero to the cell cortex $(M_i^a M_i^b)$. The attachment of the spring on the cortex is defined such that the distance between the vertices and the cortex is minimal. The resulting energy created by the action of the cortex on the nuclei is defined as follows:

$$E_{cable-to-nuclei} = \sum_{i=1}^{N} \sum_{k=1}^{N_p} \frac{1}{2} k_i^c \left(\frac{|X_i^k P_{(M_i^a M_i^b)}(X_i^k)|}{R_i}\right)^2,$$

where $P_{(D)}(Z)$ is the projection of Z on the straight line (D) and k_i^c is the stiffness of the energy.

Constraints

To ensure the biological relevance of the model, some interactions between the different agents must be introduced. For example, it is crucial for the integrity of the tissue that the nuclei stay inside the elastic box. The interactions considered are various, including nuclei/nuclei interactions, nuclei/tissue interactions, nuclei/cable interactions, etc. These interactions are modeled by dimensionless inequality or equality constraints. To simplify the notations, the argument of the constraint functions are the polygon vertices X and the box vertices Y. We list the constraints considered in the model below.

- Nuclei/nuclei non-overlapping constraint φ^p : the constraint considered has the following form:

$$\varphi_{i_1,k,i_2}^p(X) \le 0, \quad \forall (i_1, i_2) \in [1,N]^2, k \in [1,N_p],$$

where φ_{i_1,k,i_2}^p is defined later. Let us consider two cells i_1 and i_2 and one vertex k of the nucleus i_1. We denote by k^* the vertex of the polygon i_2 such that the quantity

$|d_{(X_{i_1}X_{i_2})}(X_{i_1}^k) - d_{(X_{i_1}X_{i_2})}(X_{i_2}^{k^*})|$ is minimal, with $d_{(D)}(Z)$ being the distance between the point Z and the straight line (D). Since there might be two solutions k_1^* and k_2^*, we choose k^* such that the distance between $X_{i_1}^k$ and $X_{i_2}^{k^*}$ is minimal. The expression of k^* is determined as follows:

$$k^* = argmin_{l\in[1,N_p],|X_{i_1}^k X_{i_2}^l|\leq|X_{i_1}X_{i_2}|}|d_{(X_{i_1}X_{i_2})}(X_{i_1}^k) - d_{(X_{i_1}X_{i_2})}(X_{i_2}^l)|.$$

Then the expression of the non-overlapping constraint is given by:

$$\varphi_{i_1,k,i_2}^p(X) = |X_{i_1}P_{(X_{i_1}X_{i_2})}(X_{i_1}^k)| + |X_{i_2}P_{(X_{i_1}X_{i_2})}(X_{i_2}^{k^*})| - |X_{i_1}X_{i_2}|.$$

- Nuclei/tissue non-overlapping constraint φ^t: to ensure the integrity of the tissue, it is necessary that the nuclei remain within the tissue. For this reason, we impose a non-overlapping constraint between the nuclei and the edges of the box. The non-overlapping between tissue and nuclei is expressed as follows:

$$\varphi_{i,k,j}^t(X,Y) \leq 0, \quad \forall i \in [1,N], k \in [1,N_p], j \in [1,4].$$

where $\varphi_{i,k,j}^t$ is defined by

$$\varphi_{i,k,j}^t(X,Y) = \frac{X_i^k X_i^{k-}}{|X_i^k X_i^{k-}|} \wedge \frac{Y_j X_i^k}{|Y_j X_i^k|}, \quad \forall i \in [1,N], k \in [1,N_p], j \in [1,4],$$

with $k_\pm \equiv k \pm 1 \; [N_p]$.

- Box constraints $\varphi^{b_1}, \varphi^{b_2}$: we choose to restrict the box movement by fixing the left bottom vertex of the box Y_4 to a given value $Y_0 = (x_0, y_0)$, and by fixing the vertical coordinate of the right bottom vertex of the box Y_3 to the value y_0. With this last constraint, the point Y_3 is able to slide on the horizontal axis given by $y = y_0$. The two constraints are calculated as follows:

$$\varphi_1^b(Y) = |Y_4 - Y_0| = 0 \quad \text{and} \quad \varphi_2^b(Y) = |y_3 - y_0| = 0.$$

- Surface constraint φ^s: while at a fixed time the nucleus can deform, its volume remains constant. Since the model is two-dimensional, the volume constraint translates into a surface constraint. Thus, we introduce a surface constraint intended to maintain the surface of a cell i to a surface S_i^0. The surface constraint is expressed as follows:

$$\varphi_i^s(X) = \frac{S_i - S_0^i}{S_0^i} = 0 \quad \forall i \in [1,N].$$

- Convexity constraint φ^c: the last constraint we impose on the system is the convexity of nuclei. The expression of the convexity constraint is calculated as follows:

$$\varphi_{i,k}^c(X) = \frac{X_i^k X_i^{k+}}{\left|X_i^k X_i^{k+}\right|} \cdot \frac{X_i^k X_i^{k-}}{\left|X_i^k X_i^{k-}\right|} \leq 0 \quad \forall i \in [1,N], k \in \left[1,N_p\right],$$

for $i \in [1,N]$, $k \in [1,N_p]$ and $k_\pm \equiv k \pm 1 \left[N_p\right]$.

Minimisation problem

At a given time of development, the system composed of the tissue and the nuclei is the solution of a minimisation problem. Therefore, the pseudostratified epithelium is described at all times by a minimal energy state. The variables of the minimisation problem are the polygon vertices X, representing the nuclei, the box vertices Y, representing the tissue, and the basal anchor points M^b. The potential W: $(\mathbb{R}^2)^{N\times N_p} \times (\mathbb{R}^2)^4 \times (\mathbb{R}^2)^N \longrightarrow \mathbb{R}$ created by the different energies of the system is defined by

$$W(X,Y,M^b) = E_{tissue}(Y) + E_{nuclei}(X) + E_{cable-to-nuclei}(X,Y,M^b).$$

The constraints applied to the system are the nuclei/nuclei non-overlapping constraints, nuclei/box non-overlapping constraints, box constraints, surface constraints, and convexity constraints. To simplify the notation, we consider that the inequality constraints and the equality constraints are expressed as follows:

$$\varphi^{v} \leq 0, \quad \forall v \in \{p, t, b_2, c\}, \quad \text{and} \quad \varphi^{v} = 0, \quad \forall v \in \{b_1, s\}.$$

We define by Q the set of admissible configurations of the system:

$$Q = \{(X, Y, M^b) \in (\mathbb{R}^2)^{N\times N_p} \times (\mathbb{R}^2)^4 \times (\mathbb{R}^2)^N | \varphi^p(X) \leq 0, \varphi^t(X, Y) \leq 0, \\ \varphi^{b_1}(Y) = 0, \varphi^{b_2}(Y) \leq 0, \varphi^s(X) = 0, \varphi^c(X) \leq 0\}.$$

The minimisation problem is formulated as follows: find $(\overline{X}, \overline{Y}, \overline{M^b}) \in (\mathbb{R}^2)^{N\times N_p} \times (\mathbb{R}^2)^4 \times (\mathbb{R}^2)^N$such that

$$(\overline{X}, \overline{Y}, \overline{M^b},) = argmin_{(X,Y)\in Q} W(X, Y, M^b).$$

The potential W and the constraint functions φ^n, φ^t, φ^b, φ^s, φ^c are continuous but are not all necessarily convex. Therefore, the solution of the minimisation problem $(\overline{X}, \overline{Y})$may not be unique. We consider the Lagrangian $\mathcal{L}: (\overline{X}, \overline{Y}, \overline{M^b}) \in (\mathbb{R}^2)^{N\times N_p} \times (\mathbb{R}^2)^4 \times (\mathbb{R}^2)^N \to \mathbb{R}$ associated with the minimisation problem which is defined as follows:

$$\mathcal{L}(X, Y, M^b) = W(X, Y, M^b) + \lambda^p\varphi^p + \lambda^t\varphi^t + \lambda^{b_1}\varphi^{b_1} + \lambda^{b_2}\varphi^{b_2} + \lambda^s\varphi^s + \lambda^c\varphi^c,$$

where $\lambda^p = (\lambda^p_{i_1,k,i_2})_{(i_1,k,i_2)\in[i,N]\times[1,N_p]\times[1,N]}$, $\lambda^t = (\lambda^t_{i,k,j})_{(i,k,j)\in[i,N]\times[1,N_p]\times[1,4]}$, $\lambda^b = (\lambda^b_1, \lambda^b_2)$, $\lambda^s = (\lambda^s_i)_{i\in[1,N]}$, and $\lambda^c = (\lambda^s_{i,k})_{(i,k)\in[1,N]\times[1,N_p]}$ are the Lagrangian multipliers associated with the nuclei/nuclei non-overlapping constraints, nuclei/box non-overlapping constraints, box constraints, surface constraints, and convexity constraints, respectively. The notation $\lambda^v\varphi^v$ for $v \in \{p, t, b_1, b_2, s, c\}$ implicitly considers the element-by-element product. For example, for the surface constraint, $\lambda^s\varphi^s = \sum_{i=1}^{N} \lambda^s_i\, \varphi^s_i$.

Minimisation algorithm

The resolution of non-convex minimisation problems with constraints is not trivial. Because the system is not convex, a multitude of minima may exist. Note that our aim is not to find a global minimum of the problem but rather a local minimum. Indeed, in the system the actors seek to achieve the local optimum configuration closest to their initial configuration. The algorithm used to solve the minimisation problem is named the Damped Arrow-Hurwicz Algorithm (DAHA)[S2]. It is a modification of the classical Arrow-Hurwicz Algorithm with the addition of a damping term to ensure the convergence of the algorithm.

The DAHA algorithm is an iterative algorithm. The paramter τ denotes the index of the iteration. Knowing all previous iterates, the new iterate $\tau + 1$ is calculated as follows:

$$\begin{cases} {\lambda^p_{i_1,k,i_2}}^{\tau+1} = \max(0, {\lambda^p_{i_1,k,i_2}}^{\tau} + \beta^p\varphi^p_{i_1,k,i_2}(X^\tau)), \quad \forall(i_1, k, i_2) \in [1, N] \times [1, N_p] \times [1, N], \\ {\lambda^t_{i,k,j}}^{\tau+1} = \max(0, {\lambda^t_{i,k,j}}^{\tau} + \beta^t\varphi^t_{i,k,j}(X^\tau, Y^\tau)), \quad \forall(i, k, j) \in [1, N] \times [1, N_p] \times [1,4], \\ {\lambda^{b_1}}^{\tau+1} = {\lambda^{b_1}}^{\tau} + \beta^{b_1}|\varphi^{b_1}(Y^\tau)|, \\ {\lambda^{b_2}}^{\tau+1} = \max(0, {\lambda^{b_2}}^{\tau} + \beta^{b_2}\varphi^{b_2}(Y^\tau)), \\ \lambda^{s\,\tau+1}_i = \lambda^{s\,\tau}_i + \beta^s|\varphi^s_i(X^\tau)|, \quad \forall i \in [1, N], \\ {\lambda^c_{i,k}}^{\tau+1} = \max(0, {\lambda^c_{i,k}}^{\tau} + \beta^c\varphi^c_{i,k}(X^\tau)), \quad \forall i \in [1, N], k \in [1, N_p], \end{cases}$$

and

$$
\begin{cases}
Y_j^{\tau+1} = \frac{1}{1+{c_Y}/{2}}\left(2Y_j^{\tau} - \left(1 - {c_Y}/{2}\right)Y_j^{\tau-1}\right) - \frac{\alpha_Y^2}{1+{c_Y}/{2}}\nabla_{Y_j}\mathcal{L}\left(X^\tau, Y^\tau, M_k^{b^\tau}\right) \\
\quad - \frac{\gamma^{Y,t}}{1+{c_Y}/{2}} \sum_{\substack{(i,k)\in[1,N]\times[1,N_p] \\ j\in[1,4]}} \varphi_{i,k,j}^t(X^\tau,Y^\tau)\lambda_{i,k,j}^t \nabla_{Y_j}\varphi_{i,k,j}^t(X^\tau,Y^\tau) \\
\quad - \frac{\gamma^{Y,b_1}}{1+{c_Y}/{2}}\varphi^{b_1}(Y^\tau)\lambda^{b_1}\nabla_{Y_j}\varphi^{b_1}(Y^\tau) \\
\quad - \frac{\gamma^{Y,b_2}}{1+{c_Y}/{2}}\varphi^{b_2}(Y^\tau)\lambda^{b_2}\nabla_{Y_j}\varphi^{b_2}(Y^\tau), \forall\, \mathrm{j} \in [1,4], \\
M_k^{b^{\tau+1}} = \frac{1}{1+{c_M}/{2}}\left(2M_k^{b^\tau} - \left(1 - {c_Y}/{2}\right)M_k^{b^{\tau-1}}\right) - \frac{\alpha_M^2}{1+{c_M}/{2}}\nabla_{M_k^b}\mathcal{L}\left(X^\tau, Y^\tau, M_k^{b^\tau}\right) \\
X_k^{i^{\tau+1}} = \frac{1}{1+{c_X}/{2}}\left(2X_k^{i^\tau} - \left(1 - {c_X}/{2}\right)X_k^{i^{\tau-1}}\right) - \frac{\alpha_X^2}{1+{c_Y}/{2}}\nabla_{X_k^i}\mathcal{L}\left(X^\tau, Y^\tau, M_k^{b^\tau}\right) \\
\quad - \frac{\gamma^{X,p}}{1+{c_X}/{2}} \sum_{\substack{(i_1,i_2)\in[1,N]^2 \\ k\in[1,N_p]}} \varphi_{i_1,k,i_2}^p(X^\tau)\lambda_{i_1,k,i_2}^p \nabla_{X_k^i}\varphi_{i_1,k,i_2}^p(X^\tau) \\
\quad - \frac{\gamma^{X,t}}{1+{c_X}/{2}} \sum_{\substack{(i,k)\in[1,N]\times[1,N_p] \\ j\in[1,4]}} \varphi_{i,k,j}^t(X^\tau,Y^\tau)\lambda_{i,k,j}^t \nabla_{X_k^i}\varphi_{i,k,j}^t(X^\tau,Y^\tau) \\
\quad - \frac{\gamma^{X,s}}{1+{c_X}/{2}} \sum_{i\in[1,N]} \varphi_i^s(X^\tau)\lambda_i^s \nabla_{X_k^i}\varphi_i^s(X^\tau) \\
\quad - \frac{\gamma^{X,c}}{1+{c_X}/{2}} \sum_{(i,k)\in[1,N]\times[1,N_p]} \varphi_{i,k}^c(X^\tau)\lambda_{i,k}^c \nabla_{X_k^i}\varphi_{i,k}^c(X^\tau), \forall\, \mathrm{i} \in [1,\mathrm{N}], \mathrm{j} \in [1, N_p],
\end{cases}
$$

where $\alpha_Y, \alpha_X, \alpha_M$ are parameters that control the actualisation of the variables X, Y and M^b, β^p, β^t, $\beta^b = (\beta^{b_1}, \beta^{b_2})$, β^s and β^c are parameters that control the actualisation of the Lagrangian multipliers and $\gamma^{Y,t}$, γ^{Y,b_1}, γ^{Y,b_2}, $\gamma^{X,p}$, $\gamma^{X,t}$, $\gamma^{X,c}$, $\gamma^{X,s}$, c_Y, c_X and c_M are actualisation parameters.

The stopping criterion of the minimisation algorithm is determined by

$$\frac{\mathcal{L}(X^{\tau+1}, Y^{\tau+1}) - \mathcal{L}(X^\tau, Y^\tau)}{\mathcal{L}(X^\tau, Y^\tau)} \leq \epsilon,$$

where $\epsilon > 0$ is the tolerance. This condition ensured that the Lagrangian variations were small and therefore that the minimum configuration of the system is close enough, depending on the threshold ϵ. We fix $\epsilon = 10^{-5}$.

The parameters α are related to the speed of actualisation of the position of the polygons and the box vertices in the opposite direction of the gradient of the Lagrangian (and therefore of the potential W). The speeds of actualisation of the Lagrangian multipliers associated with the constraints are controlled by the parameters β. The parameters γ control the weight of the constraints in the Lagrangian. In the initial version of the DAHA[S2], the parameters γ are

calculated using $\gamma = \alpha\beta$. However, it has been observed by the authors that considering γ independent of α and β leads to faster convergence results. It explains why γ are full-fledged parameters in this model. The parameters c are related to the damping term. In accordance with [2] we fixed $c_X = c_Y = c_{M^b} = 2$ because it has been observed that it provides better results. The values of α, β, and γ listed below have been chosen to ensure a rapid convergence of the system to the minimal energy state.

	X	Y	M^b
α	0.1	0.1	0.1
c	2	2	2

	φ^p	φ^t	φ^{b_1}	φ^{b_2}	φ^s	φ^c
β	0.1	0.1	1	1	0.01	0.1
γ	0.01	0.5	1	1	1	0.01

Time-dependent model: the cell cycle

We introduce the time dependency part of the model. During the development of the tissue, cells are subject to the cell cycle, which describes the process that cells undergo to divide. It is composed of four phases: G_1, S, G_2, and M. Let us consider the time $t \in [0, T]$ with $T > 0$. We introduced a time discretization $(t^n)_{n\in[0,N_t]}$ of $[0, T]$ with $t^n = t^{n-1} + dt$ where $dt > 0$. At each time step t^n, a cell i is in a phase of the cell cycle, namely G_1, S, G_2, or M. In the model the M phase is divided into 3 steps, while the other phases are described by one step each. Accordingly, the total number of steps in the model is 6 6 (see Figure 2D). The various steps are described in the following paragraphs.

- Steps 0 (equivalent to G_1): the phase 0 is characterised by an apical-to-basal movement of the nuclei along their cortex. In the fish retina[S3] and mouse brain[S4] this motion is considered passive. It is a consequence of other cells going through the cell cycle pushing neighbouring cells away to reach the apical surface, sending new daughter cells inside the depth of the tissue. In the model, this phase was therefore characterized by passive motion.
- Steps 1 (equivalent to S): for simplicity we decided to limit the growth of the nucleus to this phase. When a cell enters this phase at time t^{n^*}, a clock $C_i^{n_*}$ is defined to determine the time the nucleus remains in S. In the model, the increase of the volume is given by an increase in surface area. Let us consider a nucleus i that enters phase 1 at time $t_* = t^{n_*}$. The number of iterations in which the nucleus has to double its volume is given by $\overline{n}_i = \frac{\lfloor C_i^{n_*} \rfloor}{dt}$. The increase of surface area is then calculated as follows:
$$S_i^n = \pi({R_i^{n_*}}^3 + \frac{n-n^*}{\overline{n}_i}{R_i^{n_*}}^3)^{2/3}.$$
- Steps 2 (equivalent to G_2): phase nuclei migrate towards the apical region. We suppose that this motion is active[S3-S5]. The implementation of the active movement is made via the addition of an energy E_{IKNM} during the minimisation. At the cell level, apical movement is executed by the action of actomyosin[S6,S7]. We chose to model this action with a spring linking the centre of mass of the nucleus to the apical point of its

cortex. During G_2, the rest length of the spring is fixed to zero. Such a configuration induces the drag of the nuclei towards the apical region. Therefore, the energy E_{IKNM} is calculated as follows:

$$E_{IKNM}(X) = \sum_{i\in[1,N_p],P_i=2} \frac{1}{\zeta} k^{IKNM} \left(\frac{|X_i - M_i^a|}{\tau_2^s}\right)^{\zeta},$$

where k^{IKNM} is the stiffness of the spring and zeta is a parameter linked to the type of spring. Fixing $\zeta = 1$ induces the force to be constant while $\zeta = 2$ describes a hookean spring. To simulate the fact that the upward movement is not continuous in vivo[S3-S5], we define the apical-ward motion as a processive mechanism whereby the underlying motor can engage and disengage. In the model, we considered that this mechanism takes some time to organise and can also break. Let p_{IKNM}^{on} and p_{IKNM}^{off} denote the probability of engaging and disengaging the motor driving apical-ward movement, respectively. The deacrease of $p_{IKNM}^{on}$and the increase of p_{IKNM}^{off} then slow down the active motion in G_2. The creation of the new energy in the minimisation causes the introduction of another energy into the system. So far, all the movements associated with the minimisation are internal and independent of time. However, the apical-ward movement energy produces a time-dependent process that takes place over many time iterations. In the absence of other constraints, a lone nucleus would be able to cross the whole depth of the tissue in one iteration. To prevent this, we introduced a gradient flow energy. This energy adds a weight to the movement of the nucleus. At each time step t^n, the energy E_{GF} is expressed as follows:

$$E_{GF}(X) = \sum_{i\in[1,N_p]} \frac{1}{2} k^{GF} \left(\frac{|X_i - X_i^n|}{X_i^n}\right)^2,$$

with k^{GF} as the stiffness of the energy.

Once the nucleus i is close enough to the apical surface, i.e. $\left|d_{(Y_1Y_2)}(X_i) - \epsilon^{G_2}\right| \leq 0$ with $\epsilon^{G_2} > 0$ as a small threshold, the nucleus enters mitosis. Mitosis is the fastest phase of the cell cycle. However, in the model we split this phase in three steps, are described as follows:

1. Steps 3: Nuclei undergo mitosis in a narrow apical zone where only dividing nuclei can enter[S6]. There, nuclei round up, pushing neighbors away[S8,S9] . During this phase the apical stiffness energy k_i^a is set to zero. In this region, nuclei are located above all the other nuclei and, being free of pressure, become spherical. In this subphase, we set the stiffness of the cable energy to zero $k_i^c = 0$.
2. Steps 4 of the model corresponds to actual mitosis. Since this process is fast compared to the duration of the cell cycle, we considered this step to happen in one iteration. Let us consider a nucleus i in phase 4 at time t^n and its two daughter nuclei of indices i_1 and i_2. The new daughters are characterised by the surface $S_{i_1}^{n+1}$ and $S_{i_2}^{n+1}$, both of which are equal to half the surface of the mother nucleus S_i^n. It is known that nuclei divide perpendicular to the apical plane. To satisfy this condition, the position of the daughter cell is computed with the following steps:
   Let N^* be the floor value of $\frac{N_p}{2}$. Find the indices l_0 such that $[X_i^l, X_i^{l+N^*}]$ is as perpendicular as possible to the apical surface $[Y_1, Y_2]$, i.e.,
   $$l_0 = argmin_{l\in[1,N^*]} X_i^l X_i^{l+N^*} \cdot Y_0 Y_1$$
   a. Define the position of the first N^* vertices of the polygons representing the two daughter nuclei:

$$X_{i_1}^{l} = X_i^{l_0-1+l} \quad \text{and} \quad X_{i_2}^{l} = X_i^{l_0+N^*-1+l}, \quad \forall l \in [1, N^*].$$

b. Complete the definition of the last N^* vertices of the polygons representing the two daughter nuclei:

$$X_{i_1}^{l} = X_i^{l_0+N^*-1} + \left(\frac{l-N^*}{N^*+2}\right) * \left(X_i^{l_0} - X_i^{l_0+N^*}\right), \quad \forall l \in [N^*, N_p]$$

and

$$X_{i_2}^{l} = X_i^{l_0-1} + \left(\frac{l-N^*}{N^*+2}\right) * \left(X_i^{l_0+N^*} - X_i^{l_0-1}\right), \quad \forall l \in [N^*, N_p].$$

The different steps of the definition of the daughter nuclei are represented in Fig. A2. Note that to store the value of the new cells, one of the daughter nuclei is stored in the place of its mother, while the other one is created as a new nucleus.

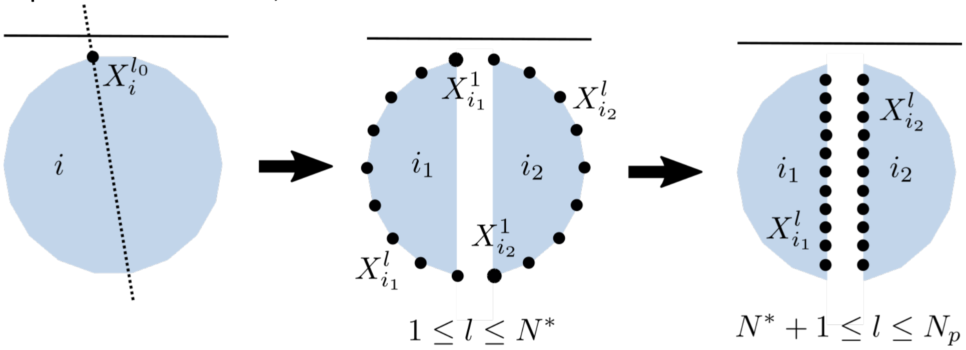

Fig. A2: Representation of the different steps leading to the division of a nucleus i. Left: Step a; Middle: Step b; Right: Step c.

3. Steps 5: The last phase of the model is related to construction of the cable (cell cortex) of the two new daughter nuclei. One of the daughter nuclei will keep the cable of its mother[S10]. This nucleus then enters phase 0. The other nucleus i_2 will enter phase 5 to be given time to construct its cortex. The average time T_3 spent by the nucleus in this phase is considered to be equal to 12 minutes. The creation of the cortex associated with nucleus i_2 is made to ensure that the cables are ordered and do not cross. The new cortex anchor points $M_{i_1}^a$ and $M_{i_1}^b$ are chosen randomly in the intervals

$$I^a = \left[P_{(Y_1Y_2)}(X_{i_2}) - \nu, P_{(Y_1Y_2)}(X_{i_2}) + \nu\right] \cap \left[M_{j_-}^a, M_{j_+}^a\right],$$

and

$$I^b = \left[P_{(Y_3Y_4)}(X_{i_2}) - \nu, P_{(Y_3Y_4)}(X_{i_2}) + \nu\right] \cap \left[M_{j_-}^b, M_{j_+}^b\right],$$

respectively, with j_- and j_+ as the indices defining the nuclei directly to the left and right of the nucleus i_2. The parameter ν is given by $\nu = 2^k R_{i_2}$, with k as the smallest integer such that the sets I^a and $I^b$are not empty.

This concludes the description of the cell cycle phases. The duration of theses phases are denoted by $T_0, T_1, T_2, T_3, T_4, T_5$ respectively. In addition, we consider the growth of the elastic box. For the sake of simplicity, the vertical dimension of the box (along the apical-basal axis) is fixed and set to provide sufficient space for multiple layers to form. This assumes that the basal surface does not constrain growth (as suggested by Fig.1B) although we cannot exclude the possibility that actin accumulation could impair the free mouvement of nuclei in this region[S1]. In contrast, we expect the lateral sides of the box to constrain growth. This is represented by allowing elastic deformation of the box perpendicular to the apical-basal axis. To allow progressive expansion of the box, at a given time step t^n, the lateral rest length of

the spring of the elastic box is updated $\tau_J^{s\,n+1} = |\overline{Y_J^{n+1}} - \overline{Y_{J+}^{n+1}}|$ with $\bar{Y}$ the solution of the minimisation problem and J the indices corresponding to the lateral spring.

Choice of the model parameters

To finish the presentation of the model we discuss the choice of its parameters. The aim of this project is to reproduce the development of the imaginal disc of Drosophila. Therefore, whenever possible, parameter values are chosen in accordance with experimental results. However, in some situations, it was difficult to relate parameters to actual data, therefore, some approximations had to be made.

To initialise the model we considered data given from [S11]. This shows that at $t = 36h$, around 70% of the cells are in the S phase, 30% in G_1, and 0% in G_2. In the model we decided to start with a small number of nuclei $N = 10$ at a developmental time of $60h$. We initialised the model with seven nuclei in the S phase and three nuclei in G_1. The initialisation of the positions of the nuclei and the elastic box are made to match the configuration of the imaginal disc of Drosophila. We initialised the nuclei and box as follows:

- The tissue was initialised as a rectangular box. The vertices of the box are defined as follows: $Y_1 = (-5, 4)$, $Y_2 = (5, 4)$, $Y_3 = (5, -4)$, and $Y_4 = (-5, -4)$.
- The nuclei were initialised on the horizontal line of coordinate $y = 0.5$ such that the nuclei were spread uniformly on this axis. This position was then perturbed by a small noise. For all $i \in N^{ini}$, the position of the vertices of the polygons are given as follows:

  $$X_i^l = X_i + R_i \begin{pmatrix} \cos(\frac{2l\pi}{N_p}) \\ \sin(\frac{2l\pi}{N_p}) \end{pmatrix} \; \forall l \in [1, N_p], \text{with } X_i = (-4.5 + i - 1.,0.5) + 0.5 R_i \epsilon_X$$

  with X_i as the position of the center of mass of the polygon and ϵ_X as a random number between zero and one chosen according to a uniform law. The radius R_i depends of the cell cycle phase the nuclei i is into. If the nuclei i is in phase G_1, then $R_i = 0.5$. For the rest of the annexwe call this value $R^{ini} = 0.5$. If the nuclei i is in phase S, R_i is chosen randomly between R^{ini} and $2^{2/3} R^{ini}$. It models the distribution of the nuclei in phase S. The number of vertices of the polygon is fixed to $N_p = 20$. This parameter is chosen to be large enough to observe the deformation and the motion of the nuclei but small enough to avoid large computational times.

In order to be able to observe phenomena with a duration of less than an hour we choose as a time step $dt = \frac{1}{10} h = 6\ min$. In addition to the information on the distribution of the nuclei in G_1, S, and G_2 during development of the imaginal disc of Drosophila, the reference [S11] also gives us information about the duration of the cell cycle phases as a function of the development time.

As previously mentioned, T_0, T_1, T_2, T_3, T_4 and T_5 are the average durations of the different steps of the model. Notice that these durations can depend on time. The choice of these durations (except for T_2, which is an output of the model) has been made with information presented in [S11] as outlined next:

- We observed that the average duration of G_1 increases over time. With a basic regression, we choose $T_0(t) = (\frac{t}{24})^2$ with t as the time in hours.

- The duration of the S phase seems to be roughly constant. We choose $T_1(t) = 8h$.
- The disengagement of the cortex action on a nucleus that has reached the apical surface is considered to happen over a short period of time (within a few minutes). Therefore, we have $T_3(t) = dt + 2\epsilon_3 dt$ with ϵ_3 as a random number between zero and one chosen according to a uniform law.
- The division of the nuclei is assumed to be instantaneous. Therefore, $T_4(t) = 0$.
- The construction of the new cortex is considered to take a few minutes, meaning that $T_5(t) = dt + 2\epsilon_5 dt$ with ϵ_5 as a random number between zero and one chosen according to a uniform law.
-

The other parameters that need to be defined are p_{IKNM}^{on} and p_{IKNM}^{off}. These two parameters provide the probability of engagement of a given nucleus with the machinery that drags it towards the apical surface. We consider that these mechanisms follow Poisson processes of parameters ν^{on} and ν^{off}, respectively. This means that the probability of starting and stopping the mechanism during a time interval dt can be approximated by

$$p_{IKNM}^{on} = 1 - e^{\nu^{on} dt} \quad and \quad p_{IKNM}^{off} = 1 - e^{\nu^{off} dt},$$

provided that dt is so small that $\nu^{on} dt \ll 1$ and $\nu^{off} dt \ll 1$. We assumed that motor engagement occurs frequently, 10 times an hour. Therefore, $\nu^{on} = 10\, h^{-1}$. To ensure that the global movement of the nuclei was oriented towards the apical membrane, we determined that the probability to disengage must be smaller than the probability to engage the motor. However, if nuclei were prevented from moving because of other nuclei, we supposed that the probability to disengage would become larger. We defined t^{IKNM} as the time in which the nuclei position has moved by a distance smaller than $0.5R^{ini}$. We then choose

$$\nu^{off} = \overline{\nu}^{off}\left(1 + \frac{t^{IKNM}}{t_0^{IKNM}}\right),$$

with $\overline{\nu}^{off} = 1\, h^{-1}$ as the frequency of the event when the nucleus is moving and t_0^{IKNM} as the time it takes for a nucleus to reach the apical membrane without any exterior constraint. This time is expected to be between 30 minutes and 1 hour. In this case, we choose $t_0^{IKNM} = 1$ h.

The last parameters to define are the ones related to the energies. They are defined as follow:

- We first considered the energies related to the elastic box. We considered the springs and torques of the box to be of the same order as those related to the nuclei and cell cortex. We then chose $k^s = 1$ and $k^t = 1$. The rest length and rest angles were chosen such that the equilibrium position is that at initialisation. This means that $l_1^s = l_3^s = 10$, $l_2^s = l_4^s = 8$ and $\tau_j^t = \pi/2$ for $j \in \{1,4\}$.
- The apical energy prevents nuclei from getting too close to the apical membrane. So that this energy is stronger than the one of the nuclei, we choose $k_i^a = 10\ if\ C_i \in \{0,1\}$ and $k_i^a = 0$ if $C_i \in \{2,3,4,5\}$. The thickness of the apical layer was chosen to be $l^a = 3R^{ini}$.
- The distance energy models the action of the cell cortex on nuclei while the bending energy models the preferred shape of the nuclei. Here we have chosen parameters that reproduce experimental nuclear shapes. On this basis, we have fixed $k_1^b = 0.1$ and $k^c = 0.1$.
- The IKNM energy controls the migration of the nuclei in the G_2 phase (equivalent to phase 2 in the model); therefore, for a given nucleus i, when $C_i \in \{0,1,3,4,5\}$,

$k_i^{IKNM} = 0$. When $C_i = 2$, we consider $k_i^{IKNM} = k^{IKNM}$, which does not depend on time. Given that at early stages the duration of G_2 is less than one hour, we fix $k^{IKNM} = 0.05$ and $k^{GF} = 0.01$ so that the duration for one nucleus to reach the apical membrane is between 30 minutes and 1 hour.

In the paper, each model simulation has been run for 20 initialisations. The difference between each initialisation is produced by a change of the random seed (all the other parameters are similar). For an initialisation ini the seed is defined by the following FORTRAN code:

```
call Random_seed(size = n)
allocate(seed(n))
seed = ini + 37 * (/ (i - 1, i = 1, n) /)
call Random_seed(PUT = seed)
deallocate(seed)
```

Analysis of the simulations

In this section we describe the tools used to analyse the results of the Individual-Based Model. In particular we detail the quantifiers used in this study: the number of layer, the crowding, the apical/basal position of the nuclei, the average time spent in G_2, velocity of the nuclei during the last hour in G_2. The numerical simulations have been performed in FORTRAN and we compare the results of 20 initialisations.
In the simulations, we observe an increase in the number of layers of nuclei. To quantify this increase in the numerical simulations we study the quantity

$$N_l = \frac{2RN}{(|Y_1 - Y_2| + |Y_3 - Y_4|)/2},$$

as a function of the time iteration (see Fig. 5F). As previously mentioned, N is the number of nuclei at a given time and R is the radius of a spherical nuclei in the G_1 phase. The distance $(|Y_1 - Y_2| + |Y_3 - Y_4|)/2$ represents the average length of the box, taking into account its possible deformation. The formula $\frac{|Y_1 - Y_2| + |Y_3 - Y_4|)/2}{2R}$ then corresponds to the number of nuclei organised in one layer that can fit in the length of the tissue. Therefore, N_l gives us an insight into the number of layers of nuclei present in the tissue at a given time.

The apical basal depth of the nuclei in the tissue is quantified by considering the average depth of the nuclei in the tissue $D = \frac{1}{N} \sum_{i=1}^{N} d_{(Y_1 Y_2)}(X_i)$. This quantity is presented in Fig. 3B.

Another parameter we are interested in is the crowding observed in the tissue. To quantify this increase in the simulation, we computed the crowding in a similar manner to the experimental data treatment presented in Fig. 1F with the difference that surfaces are considered instead of volumes. This quantity is plotted in Figs. 3C and 5F).

To compare the overall behaviours of the nuclei as functions of space we consider the distribution of the nuclei in phases G_1, S, and G_2 along the A-B axis (see Figs. 3G and 5B). In the simulations, the apical/basal axis was discretised in small subsets of length $h = 0.2R^{ini}$. For each subset, the number of nuclei in G_1, S, and G_2 were computed. We then had the

number of nuclei in each of the phases relative to the position on the basal/apical axis. This value can be reformulated to obtain the number of nuclei in each of the phases as a function of the apical distance.
Finally we are interesting in the evolution of the nuclei during G_2. Let $it_{G_2}^{in}(i)$ and $it_{G_2}^{out}(i)$ be the iteration number corresponding to the time a nucleus i enters and leaves G_2, respectively. Then we can compute the average time $T_{G_2}^{in/out}$ needed for a nucleus that has entered G_2 to reach the apical membrane. $T_{G_2}^{in/out}(t)$ is then calculated as follows:

$$T_{G_2}^{in/out}(t) = \frac{dt}{Card(\overline{Q}_{G_2}^{it})} \sum_{i \in \overline{Q}_{G_2}^{it}} |it_{G_2}^{out}(i) - it_{G_2}^{in}(i)|,$$

with $\overline{Q}_{G_2}^{it} = \{i \in [1, N] | it_{G_2}^{in}(i) \leq it \leq it_{G_2}^{out}(i)\}$. In addition we compute the apical distance $D_{G_2}^{in/out}$ of the nuclei to the apical membrane when they enter the phase G_2:

$$D_{G_2}^{in/out}(t) = \frac{dt}{Card(\overline{Q}_{G_2}^{it})} \sum_{i \in \overline{Q}_{G_2}^{it}} |X_i^{it_{G_2}^{out}(i)} - X_i^{it_{G_2}^{in}(i)}|.$$

Finally, we consider the velocity $V_{G_2}^{out_{1h}}$ of the nuclei in the hour before their division:

$$V_{G_2}^{out_{1h}}(t) = \frac{dt}{Card(\tilde{Q}_{G_2}^{it})} \sum_{i \in \overline{Q}_{G_2}^{it}} \frac{|X_i^{it_{G_2}^{out}(i)} - X_i^{it_{G_2}^{out}(i)-1}|}{1},$$

with $\tilde{Q}_{G_2}^{it} = \{i \in [1, N] | it_{G_2}^{out}(i) - 1 \leq it \leq it_{G_2}^{out}(i)\}$.

Description of the basal mechanism

We hypothesise that the nuclei are able to transition from G_1 to S when the nuclei receive a signal from the basal membrane. We define the range of diffusion by λ.
In the model, the transition to the S phase is considered to follow a Poisson process of parameters $\nu_{G_1/S}$. This means that the probability $P_{G_1/S}$ of a nucleus in G_1 changing phase at each time step is approximated by

$$P_{G_1/S} = 1 - e^{-\nu_{G_1/S} dt},$$

provided that dt is small enough to verify $\nu_{G_1/S} dt \ll 1$. The parameter $\nu_{G_1/S}$ is idealistically computed as a function of the distance of a nucleus to the basal membrane. However, in the model, the width of the box is fixed, which is not the case in real tissue. Therefore, the probability $P_{G_1/S}$ is instead a function of the distance of a given nucleus to the nucleus that is the closest to the basal membrane at the time of its birth. At a given birth time , we denote by i_t^* the nucleus that is the closest to the basal membrane. Then the distance to the basal membrane is given by $d_b = d_{(Y_3Y_4)}(X_j) - d_{(Y_3Y_4)}(X_{i_t^*})$. We define the probability of a nucleus j to transition from G_1 to S of at time t as follows:

$$\nu_{\frac{G_1}{S}}(j, t) = 30\overline{\nu}_{G_1/S} e^{-\frac{\ln(0.1)}{\lambda} d_b} 1_{d_b \leq \lambda},$$

with $\overline{\nu}_{G_1/S}$ and λ the maximal frequency and the diffusion length, respectively. This formula means that the frequency of the transition from G_1 to S for the nuclei that are closest to the basal membrane is $\overline{\nu}_{G_1/S}$. The frequency decreases to zero for nuclei located at a distance λ from the nucleus i_t^*, meaning that the probability of entering S is equal to zero. The probability is then set to zero for the rest of the nuclei. The frequency $\overline{\nu}_{G_1/S}$ is set to 0.7 per hour, meaning that the transition of the nuclei closest to the basal membrane occurs less than

once every hour. The diffusion length varies between 2, 4 and 10 depending on the simulations.

Methods S1 References

S1. Lele, T.P., Dickinson, R.B., and Gundersen, G.G. (2018). Mechanical principles of nuclear shaping and positioning. J Cell Biol *217*, 3330-3342. 10.1083/jcb.201804052.

S2. P. Degond, M.A.F., and S. Motsch (2017). Damped arrow–hurwicz algorithm for sphere packing. Journal of Computational Physics, 332:347

S3. Leung, L., Klopper, A.V., Grill, S.W., Harris, W.A., and Norden, C. (2011). Apical migration of nuclei during G2 is a prerequisite for all nuclear motion in zebrafish neuroepithelia. Development *138*, 5003-5013. 10.1242/dev.071522.

S4. Okamoto, M., Shinoda, T., Kawaue, T., Nagasaka, A., and Miyata, T. (2014). Ferret-mouse differences in interkinetic nuclear migration and cellular densification in the neocortical ventricular zone. Neurosci Res *86*, 88-95. 10.1016/j.neures.2014.03.011.

S5. Norden, C., Young, S., Link, B.A., and Harris, W.A. (2009). Actomyosin is the main driver of interkinetic nuclear migration in the retina. Cell *138*, 1195-1208. 10.1016/j.cell.2009.06.032.

S6. Meyer, E.J., Ikmi, A., and Gibson, M.C. (2011). Interkinetic nuclear migration is a broadly conserved feature of cell division in pseudostratified epithelia. Curr Biol *21*, 485-491. 10.1016/j.cub.2011.02.002.

S7. Liang, L., Haug, J.S., Seidel, C.W., and Gibson, M.C. (2014). Functional genomic analysis of the periodic transcriptome in the developing Drosophila wing. Dev Cell *29*, 112-127. 10.1016/j.devcel.2014.02.018.

S8. Stewart, M.P., Helenius, J., Toyoda, Y., Ramanathan, S.P., Muller, D.J., and Hyman, A.A. (2011). Hydrostatic pressure and the actomyosin cortex drive mitotic cell rounding. Nature *469*, 226-230. 10.1038/nature09642.

S9. Matthews, H.K., Delabre, U., Rohn, J.L., Guck, J., Kunda, P., and Baum, B. (2012). Changes in Ect2 localization couple actomyosin-dependent cell shape changes to mitotic progression. Dev Cell *23*, 371-383. 10.1016/j.devcel.2012.06.003.

S10. Kosodo, Y., and Huttner, W.B. (2009). Basal process and cell divisions of neural progenitors in the developing brain. Dev Growth Differ *51*, 251-261. 10.1111/j.1440-169X.2009.01101.x.

S11. Wartlick, O., Mumcu, P., Kicheva, A., Bittig, T., Seum, C., Julicher, F., and Gonzalez-Gaitan, M. (2011). Dynamics of Dpp signaling and proliferation control. Science *331*, 1154-1159. 10.1126/science.1200037.
